# Hemodynamic disturbance and mTORC1 activation: Unveiling the biomechanical pathogenesis of thoracic aortic aneurysms in Marfan syndrome

**DOI:** 10.1016/j.jpha.2024.101120

**Published:** 2024-10-28

**Authors:** Ming-Yuan Liu, Meili Wang, Junjun Liu, An-Qiang Sun, Chang-Shun He, Xin Cong, Wei Kong, Wei Li

**Affiliations:** a Department of Vascular Surgery, Beijing Friendship Hospital, Capital Medical University, Beijing Center of Vascular Surgery, Beijing, 100050, China; b Department of Vascular Surgery, Peking University People's Hospital, Beijing, 100044, China; c Department of Physiology and Pathophysiology, School of Basic Medical Sciences, Capital Medical University, Beijing, 100069, China; d Department of Vascular Surgery, The Affiliated Hospital of Qingdao University, Qingdao, 266000, Shandong, China; e School of Biological Science and Medical Engineering, Beihang University, Beijing, 100083, China; f Department of Physiology and Pathophysiology, School of Basic Medical Sciences, Peking University, Beijing, 100191, China; g The Key Laboratory of Molecular Cardiovascular Science, Ministry of Education, Beijing, 100191, China

**Keywords:** Thoracic aortic aneurysm (TAA), mTORC1, Marfan syndrome, Biomechanicalpathogenesis, Wall shear stress (WSS)

## Abstract

Thoracic aortic aneurysm (TAA) significantly endangers the lives of individuals with Marfan syndrome (MFS), yet the intricacies of their biomechanical origins remain elusive. Our investigation delves into the pivotal role of hemodynamic disturbance in the pathogenesis of TAA, with a particular emphasis on the mechanistic contributions of the mammalian target of rapamycin (mTOR) signaling cascade. We uncovered that activation of the mTOR complex 1 (mTORC1) within smooth muscle cells, instigated by the oscillatory wall shear stress (OSS) that stems from disturbed flow (DF), is a catalyst for TAA progression. This revelation was corroborated through both an MFS mouse model (*Fbn1*^+/C1039G^) and clinical MFS specimens. Crucially, our research demonstrates a direct linkage between the activation of the mTORC1 pathway and the intensity in OSS. Therapeutic administration of rapamycin suppresses mTORC1 activity, leading to the attenuation of aberrant SMC behavior, reduced inflammatory infiltration, and restoration of extracellular matrix integrity—collectively decelerating TAA advancement in our mouse model. These insights posit the mTORC1 axis as a strategic target for intervention, offering a novel approach to manage TAAs in MFS and potentially pave insights for current treatment paradigms.

## Highlights

• The biomechanical mechanism of TAA remains unclear compared to physiological factors.
• Disturbed flow induced OSS activates mTORC1 pathway, leading to phenotype switch in aortic smooth muscle cells.
• This work offers biomechanical-based therapeutic insights for TAA treatment.

## Graphical abstract

**Figure:**
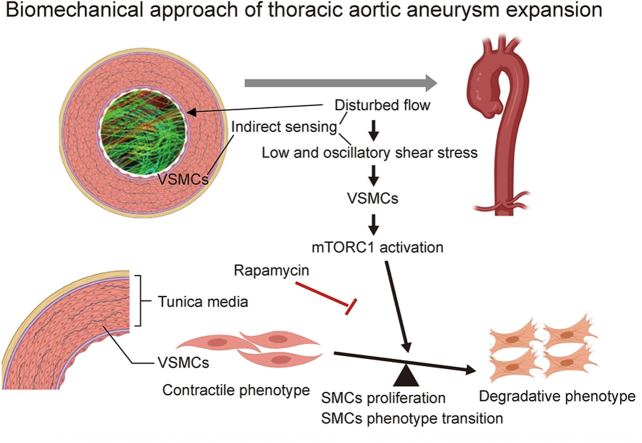
Biomechanical Approach of Thoracic Aortic Aneurysm Expansion

## 1 Introduction

Thoracic aortic aneurysm (TAA) presents a significant clinical challenge in Marfan syndrome (MFS), with current treatments primarily involving surgical interventions [[1], [2], [3]]. Despite its prevalence, the mechanisms underpinning TAA's development, especially from a biomechanical perspective, remain inadequately understood [[4], [5], [6]]. This gap hinders the advancement of effective non-surgical interventions, as current therapeutic strategies are mostly limited to surgical or endovascular repairs due to a limited grasp of TAA's pathogenesis [7].

Recent advances have shed light on the pathogenesis of TAA, emphasizing the role of biomechanical forces in its development [6,[8], [9], [10], [11]], specifically, oscillatory shear stress (OSS), in shaping the disease's trajectory [12,13]. These biomechanical stresses are known to induce structural and functional alterations within the aortic wall [7], yet the precise biochemical pathways mediating these changes have not been fully elucidated. Endothelial cells (ECs) serve as primary sensors of hemodynamic changes, particularly OSS, which is implicated in vascular pathologies. These ECs can transmit mechanical signals to the underlying smooth muscle cells (SMCs), which in turn are more responsive to intense mechanical forces and extracellular matrix (ECM) alterations [11,14]. However, the precise mechanisms by which OSS regulates aneurysm progression through EC/SMC interactions in TAA pathogenesis remain elusive [6]. This knowledge gap underscores the need for comprehensive studies that elucidate the intricate relationships between hemodynamic forces, cellular responses, and signaling pathways in the context of TAA development, particularly in conditions such as MFS where vascular integrity is compromised.

*In vitro* experiments have demonstrated that OSS can induce sustained activation of the mammalian target of rapamycin (mTOR) signaling pathway [15,16]. Interestingly, the role of mTOR is implicated in MFS due to its role in modulating SMCs behavior within the aortic media [[17], [18], [19]]. The hyperactivation of mTOR results in SMCs acquiring a degradative phenotype, which compromise the structural integrity of the aortic wall, rendering it more susceptible to aortic dilatation and dissection. Yet, there is a notable absence of *in vivo* and temporal studies to elucidate the potential pathogenesis and interplay between OSS, mTOR, and TAA enlargement in patients with MFS [20].

In light of these challenges, this study ventures into uncharted territory by elucidating the biomechanical etiology of TAA, specifically scrutinizing the role of the mTOR signaling pathway in response to disturbed flow (DF)-induced OSS. Building on the known association between OSS and vascular remodeling [[21], [22], [23]], our investigation delineates the correlation between OSS and mTOR complex 1 (mTORC1) pathway activation, hypothesizing that OSS is a pivotal mechanistic force in TAA development. Through a combination of *in vivo* modeling in *Fbn1*^+/C1039G^ mice and analysis of human MFS tissue samples, we aim to dissect the mTORC1 pathway's response to DF and its contribution to the aneurysmal architecture.

By integrating molecular insights with biomechanical stimuli, we anticipate shedding light on the pathogenetic landscape of TAA in MFS. This may recalibrate therapeutic strategies, highlighting mTORC1 inhibition as a potential target for mitigating TAA progression. Such advances bear the promise of bridging the gap between symptomatic treatment and curative therapy, offering hope for improved patient outcomes in MFS.

## 2 Materials and methods

### 2.1 Materials

Rapamycin was purchased from Selleck Chemicals (S1039; Houston, TX, USA). Antibodies against phosphorylated ribosomal protein S6 kinase (p-S6K, Thr421/Ser 424, 9204), total S6K (t-S6K, 9202), p-S6 (Ser 235/236, 4858), t-S6 (2217), phosphorylated extracellular signal-regulated kinase (*p*-ERK, 9101), and *p*-Smad 2 (18338) were purchased from Cell Signaling Technology (Boston, MA, USA). Antibodies against CD68 (ab125212), smooth muscle myosin heavy chain (SMMHC, 133567), α-smooth muscle actin (α-SMA, 108424), smooth muscle 22 (SM22, ab10135), glyceraldehyde-3-phosphate dehydrogenase (GAPDH, ab9483), and β-actin (ab119952) were purchased from Abcam (Cambridge, UK). The CD31was purchased form R&D Systems (AF3628; R&D Systems, Hong Kong, China). The NOS3 was purchased form Proteintech (27120-1-AP; Rosemont, IL, USA) and dihydroethidium (DHE) was purchased form Invitrogen (D11347; Waltham, MA, USA). The Alexa Fluor-488; 633; 594-conjugated secondary antibody was purchased from Invitrogen (Carlsbad, CA, USA). The IRDye 700DX-conjugated secondary antibody was purchased from Rockland, Inc. (Gilbertsville, PA, USA).

### 2.2 Sample collection

In this study, MFS (*n* = 6) patients undergoing elective surgical procedures for TAA between 2016 and 2019 at the Department of Vascular Surgery, Peking University People's Hospital. All human studies were performed in accordance with the Helsinki Declaration of 2013. The research protocol and informed consent for tissue collection were approved by the Ethics Committee of the Peking University People's Hospital (IRB number: 2017HB166-01). Diagnoses were made according to the Ghent nosology criteria [24]. Participants were 20–60 years old with ascending aorta aneurysms, excluding specific cardiovascular and systemic conditions. Aneurysms were defined as having a diameter >1.5 times the norm for the patient's demographics. Data were retrospectively collected on aneurysm characteristics and surgical details. Patients' ages ranged from 20 to 60 (mean = 34 years). Aorta diameters were preoperatively measured using computed tomography angiography (CTA), and thoracic aortic tissues were collected during surgery.

### 2.3 Animals

All animal experiments were conducted following the international guidelines for animal care, specifically adhering to the guidelines set forth by the NIH's Guide for the Care and Use of Laboratory Animals (NIH Publication No. 85-23, revised 1996) and received approval from the Laboratory Animal Review Board of Beijing Friendship Hospital, Capital Medical University (IRB No.20-2007). *Fbn1*^+/C1039G^ heterozygous mice, procured from Jackson Laboratory (Bar Harbor, ME, USA), harboring a mutation in the *Fbn1* gene [25], served as a validated model for MFS. Wild-type (WT) *Fbn1*^*+/+*^ littermates were utilized as controls to mitigate the effects of genetic background and pharmacological variability. To eliminate potential gender-related influences on aortic pathology, the study exclusively employed male mice, which were housed under uniform conditions of diet and hydration.

Genotypic validation of the mice was performed via polymerase chain reaction (PCR) analysis of genomic DNA. A subgroup of mice was administered rapamycin (Calbiochem, Darmstadt, Germany) at 2 mg/kg/day intraperitoneally (i.p.), while control littermates received dimethyl sulfoxide (DMSO) vehicle. The diet consisted of standard rodent chow from Research Diet, Inc. (New Brunswick, NJ, USA). Rapamycin was administered in two distinct protocols: a 22-week long-term regimen from 8 to 30 week of age (*Fbn1*^+/C1039G^ + RA, Fig. S1A) and a 4-week short-term regimen from 12 to 16 week of age (*Fbn1*^+/C1039G^ + RA(ST), Fig. S1B). At the end of the experiments, mice were humanely euthanized with an overdose of isoflurane (5%), and the aortas were harvested for subsequent analyses. In our study, a total of 64 mice were used to complete the experiments. The rapamycin treatment was conducted as a randomized, double-blind experiment. This approach ensured that neither the researchers nor the participants knew which mice were receiving the treatment or the control, thereby minimizing potential bias.

### 2.4 Color Doppler ultrasound analysis

Following anesthesia with 2% isoflurane, mice were placed on a heated pad set at 37 °C to prevent hypothermia, and hair removal was performed on the chest area. Color Doppler ultrasound examinations of the thoracic aorta were conducted at systole using the Vevo 2100 system (VisualSonics, Toronto, Canada). Parameters measured included maximal internal aortic diameter, aortic insufficiency, end-diastolic volume, and flow velocity. Two independent, blinded investigators carried out all measurements.

### 2.5 Blood pressure measurement

As detailed in previous protocols [26,27], blood pressure was measured in conscious 12-week-old mice using the tail cuff method. The mice were acclimatized to the procedure over three days, with recordings taken over the following two days. An average of more than 60 readings was computed for each mouse to derive systolic, diastolic, and pulse pressures.

### 2.6 Computational fluid dynamic (CFD) analysis

CFD analysis was conducted using three-dimensional (3D) models constructed from Digital Imaging and Communications in Medicine (DICOM) data obtained from mouse computed tomography (CT) scans. The models were analyzed using ANSYS CFX software (ANSYS, Inc., Canonsbury, PA, USA) to visualize blood flow, employing methods previously validated [28]. The Navier-Stokes equations were numerically solved, with time-averaged wall shear stress (TAWSS) and oscillatory shear stress (OSS) computed over a cardiac cycle. OSS was defined based on standard criteria, and measurements were made by two independent observers to ensure reproducibility. OSS was obtained as the overlap between regions of low wall shear stress (WSS) (<10 dyn/cm^−2^) and regions of high oscillatory WSS [12].

### 2.7 Microarray data

Microarray and RNA-seq data were acquired using the Illumina HiSeq 4000 platform provided by BGI Corporation (Shenzhen, China). Pathway analyses of differentially expressed genes (DEGs) were performed based on the Kyoto Encyclopedia of Genes and Genomes (KEGG) database, with visual representations including volcano plots and heatmaps generated on the Dr. Tom network platform (BGI). Criteria for DEG selection were *P*-value <0.05 and |log 2 fold-change| > 1.

### 2.8 En face, immunofluorescence, histochemical and DHE staining

Mouse aortas were perfused with 4% paraformaldehyde in phosphate buffered saline (PBS). After dissection, the specimens were opened, permeabilized with 0.1% Triton X-100, blocked with 1% bovine serum albumin (BSA), and then incubated with primary antibody at 4 °C overnight. The tissues were further labeled with Alexa Fluor-488-conjugated secondary antibody at 37 °C for 2 h. After three washes with PBS, the aortas were mounted on slides with mounting medium containing 4՛-6-diamidino-2-phenylindole (DAPI) (Vector Laboratories, Inc., Burlingame, CA, USA). The nonimmune IgG instead of primary antibody served as a negative control. Images were captured under a confocal microscope (Leica TCS SP8, Wetzlar, Germany). In each mouse, three images were obtained from three regions in the aortic arch and thoracic aorta. In addition, 3D confocal laser-scanning microscopy detection of the immunofluorescence and 3D rendering of the entire tunica media from the ascending aortas were established following a previously reported protocol.

The aortas were excised and embedded in OCT (Tissue Tek, Sakura Finetek, Tokyo, Japan), and 5-μm-thick sections at 70-μm intervals were mounted on slides. Sections were prepared and subsequently stained with hematoxylin and eosin (H&E; BASO Precision Optics, Ltd., Taiwan, China), elastic van Gieson (EVG; BASO Precision Optics, Ltd., Taiwan, China), and Masson's trichrome (Shanghai Bogoo Biotechnology, Shanghai, China) according to the manufacturers' instructions. For determination of the degree of elastin degradation, we applied a standard score for the grades of elastin degradation as described previously [29]: grade 1, no degradation; grade 2, mild elastin degradation; grade 3, severe elastin degradation; and grade 4, aortic rupture.

To prepare the DHE staining solution, 1 mg of DHE was dissolved in 317 μL of DMSO to create a 10 mM stock solution. The stock solution was then diluted by adding 25 μL of the stock solution to 50 mL of Milli-Q pure water, resulting in a 5 μM staining solution. Fresh 5 μM DHE staining solution was prepared in a slide box prior to use. Slides were rinsed in pure water for 30 s to remove any OCT compound. Immediately after rinsing, the slides were placed in the DHE staining solution and incubated for 5–20 min at room temperature, protected from light. After incubation, the slides were transferred to a beaker containing deionized water and washed for 1 min. The washing step was repeated twice, and the slides were kept in deionized water. Fluorescence imaging was performed immediately after the staining and washing procedure.

### 2.9 Western blot

The ascending and arch aortas were lysed in radio-immunoprecipitation assay lysis (RIPA) buffer (Beyotime, Shanghai, China) supplemented with protease and phosphatase inhibitors (Roche Diagnostics, Indianapolis, IN, USA) following a standard protocol. Briefly, equal amounts of total proteins were resolved on 10% or 12% sodium dodecyl sulfate-polyacrylamide gel electrophoresis (SDS-PAGE) gels. After transfer, the membranes were incubated with the indicated primary antibodies and IRDye 700DX-conjugated secondary antibodies. Then, the blots were visualized by an Odyssey fluorescence detection system (LI-COR Biosciences, Lincoln, NE, USA) and further quantified using NIH ImageJ software.

### 2.10 RNA isolation and quantitative reverse-transcription PCR (qRT-PCR)

Total RNA from aortas was prepared using TRIzol Reagent (Invitrogen, Waltham, IL, USA). Then, cDNA was synthesized from 1 μg of total RNA with the PrimeScript RT Reagent Kit (TaKaRa, Ohtsu, Japan). qRT-PCR was carried out by using SYBR Premix Ex Taq (TaKaRa, Ohtsu, Japan). β-actin and GAPDH were served as an internal control. The primers are shown in Table S1.

### 2.11 Statistical analysis

All data are presented as the mean ± standard error of the mean (SEM). Statistical analyses were performed using Prism 6.0 software (GraphPad Software, San Diego, CA, USA). The normality of the data distribution was determined with Kolmogorov-Smirnov tests, and all data showed a normal distribution. The detailed statistical analysis for each experiment is listed in the corresponding figure legends. Student's *t*-test was used for comparisons between two groups. One-way analysis of variance (ANOVA) was used for comparisons among more than two groups, followed by the Newman-Keuls multiple comparison test. The Mann-Whitney *U* test was used for evaluation of elastin degradation. The number of mice used for each *in vivo* analysis is indicated in the corresponding figure legends. *P* < 0.05 was considered statistically significant.

## 3 Results

### 3.1 DF in aortas augments mTOR pathway activation in MFS

**Fig. 1:**
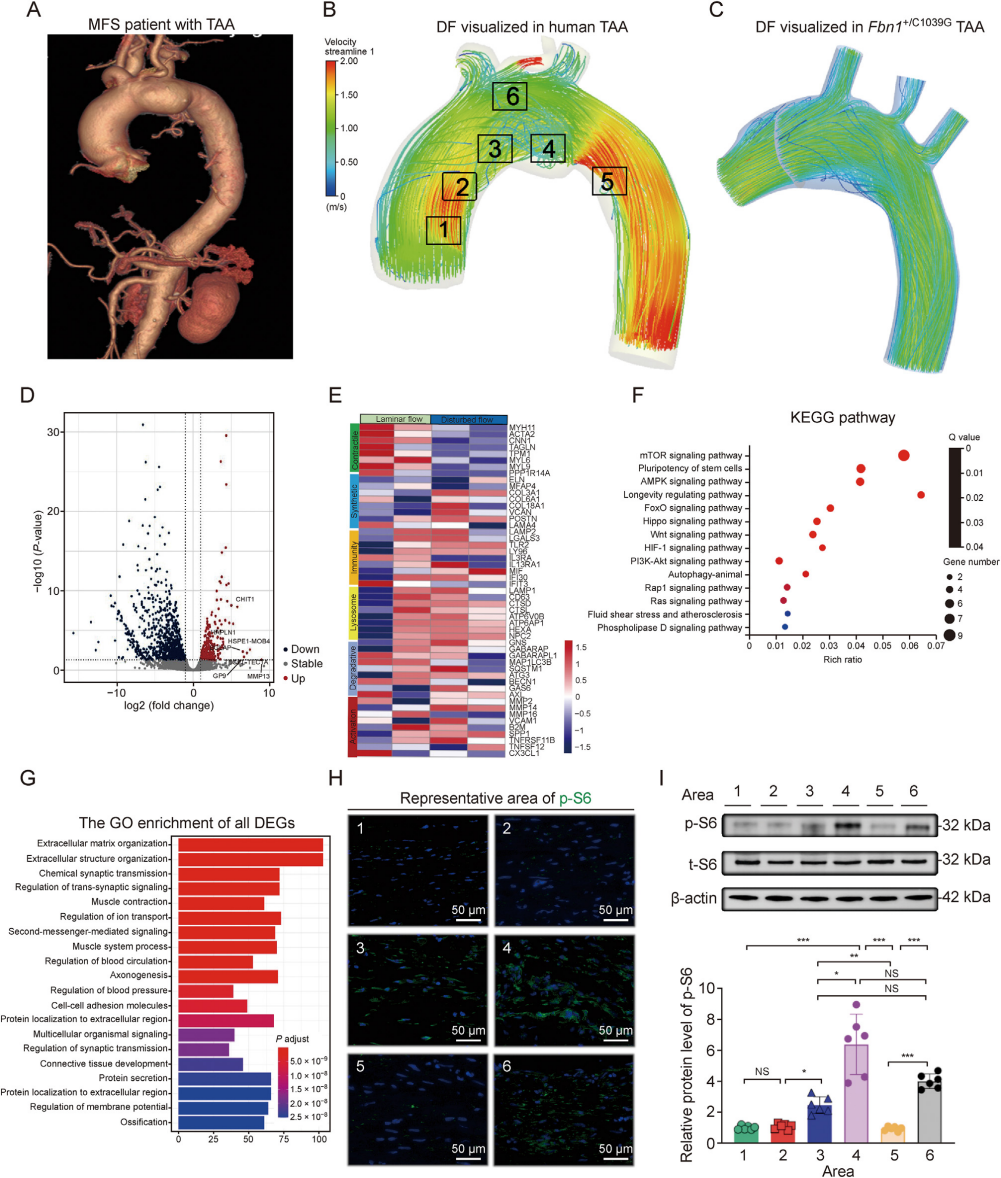
Disturbed flow (DF) significantly upregulates the phosphatidylinositol 3-kinase-akt-mammalian target of rapamycin (PI3K-Akt-mTOR) pathway in Marfan syndrome (MFS) patients. (A) Computed tomography angiography (CTA) reconstruction of a thoracic aortic aneurysm (TAA) in an MFS patient. (B, C) Computational fluid dynamic (CFD)-generated visualizations comparing DF and laminar flow (LF) in an MFS patient's TAA (B) and in a TAA of *Fbn1*^+/C1039G^ mice (C). Numbered boxes indicate representative areas under DF or LF patterns (boxes 1 and 2: DF; boxes 3 and 6: LF; boxes 4 and 5: DF). (D) A volcano plot highlights expression differences of differentially expressed genes (DEGs) between aortas under DF and LF from MFS patients, with 1,174 significant genes (260 upregulated in red, 914 downregulated in blue) between the DF (*n* = 2) and LF (*n* = 2) groups (*P* < 0.05). (E) A heatmap shows gene expression levels, with red indicating upregulation and blue indicating downregulation under LF (Control) and DF. (F) Kyoto Encyclopedia of Genes and Genomes (KEGG) pathway analysis from RNA-seq data contrasting DF with LF in MFS patient samples, where a false discovery rate (FDR) <0.01 denotes significant enrichment. (G) Gene Ontology (GO) enrichment analysis of DEGs between DF and LF. (H) Representative immunofluorescence images of phosphorylated S6 (p-S6, green) expression in the corresponding areas. (I) Representative western-blot images and analysis of P-S6 levels. Data are presented as mean ± standard error of the mean (SEM) for *n* = 6 per group. Statistical significance was determined by one-way analysis of variance (ANOVA), with ^∗^*P* < 0.05, ^∗∗^*P* < 0.01 and ^∗∗∗^*P* < 0.001, indicating significant differences; NS: not significant.

Advanced 3D phase-contrast magnetic resonance imaging (4D-flow MRI) and transcriptome analysis elucidate [12,13,30] that the aberrations in aortic hemodynamics, particularly DF and OSS, are intimately connected with aortic dilation in MFS. Our inquiry began with the visualization of aortic streamlines in both human MFS patients and mouse models, utilizing CFD analysis to reveal pronounced DF within TAA (Figs. 1A–C). Subsequent RNA-sequencing of human TAA samples, informed by CFD analysis, delineated regions of normal laminar versus DF in samples harvested from four MFS patients prior to surgery. Differential gene expression analysis unveiled a significant distinction in the transcriptomic landscape between these regions, with 260 genes upregulated and 914 downregulated in areas experiencing DF (Fig. 1D). This disparity was mirrored in phenotypic markers where vascular smooth muscle cells (VSMCs) in DF regions demonstrated a downregulation of contractile genes and an upregulation of genes associated with synthetic activity and cellular activation (Fig. 1E). KEGG pathway analysis further pinpointed the mTOR pathway as markedly enriched in regions subjected to DF, aligning with prior assertions [19] that the mTOR signalling serves as a fundamental mechanosensing pathway (Fig. 1F). This was bolstered by Gene Ontology (GO) functional enrichment analysis, which highlighted significant processual differences related to extracellular matrix dynamics and cellular mechanical response (Fig. 1G).

Considering previous researches identifying the phosphatidylinositol 3-kinase-akt-mammalian target of rapamycin (PI3K-Akt-mTOR) pathway [18,31] elevation as a therapeutic avenue in mouse models of thoracic aortic pathology, our current study ventures deeper, examining TAA samples from MFS patients across six distinct aortic regions. Consistent with our hypothesis, the expression of p-S6, indicative of mTORC1 activity, was substantially higher in regions experiencing DF both in the level of immunofluroscence and Western blot (Figs. 1H and I). Complementary histological analyses, such as EVG and Masson staining, corroborated these molecular findings with observable exacerbations in elastin degradation and medial thickening where DF prevailed (Fig. S2).

### 3.2 *In vivo* activation of the mTORC1 pathway by DF-induced OSS in MFS mouse model

**Fig. 2:**
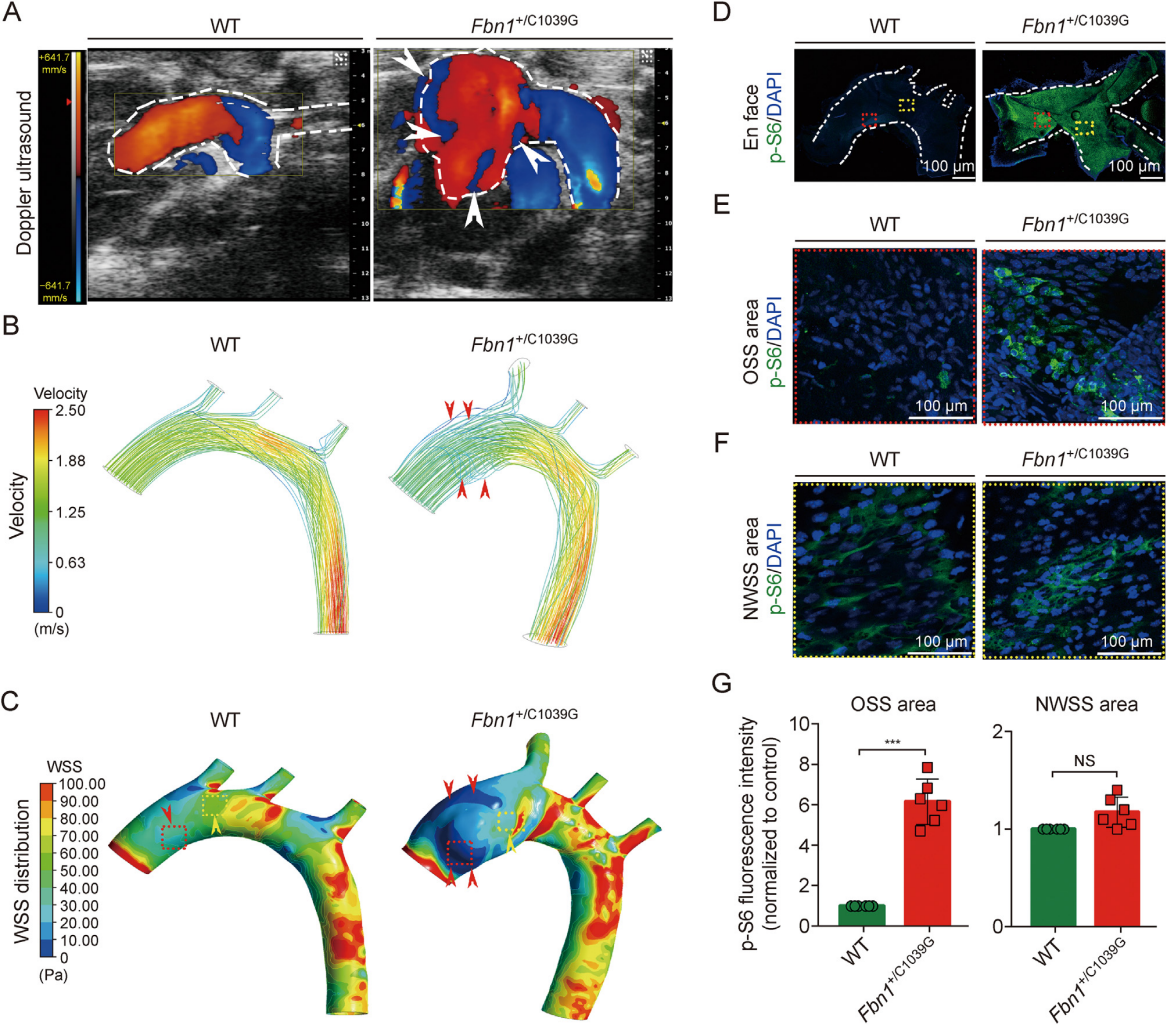
Oscillatory shear stress (OSS) promotes mechanistic target of rapamycin complex 1 (mTORC1) pathway activation in a mouse model of Marfan syndrome (MFS). (A) Representative color Doppler ultrasound images illustrate aortic flow during systole in *Fbn1*^+/+^ wild-type (WT) and *Fbn1*^+/C1039G^ mice at 30 week. Regions of oscillatory flow are indicated by white arrowheads. (B) Computational fluid dynamics (CFD) simulations visualize aortic velocity and flow streamlines, with disturbed flow patterns, indicated by red arrowheads, in both WT and *Fbn1*^+/C1039G^ mice. (C) Wall shear stress (WSS) distribution is mapped across the aorta, highlighting OSS regions, shown in dark blue and pointed out by red arrowheads. An area of representative OSS is delineated by a red dotted outline, contrasting with an area of normal WSS (NWSS) circumscribed by a yellow dotted outline. (D) En face immunofluorescence staining for phosphorylated ribosomal protein S6 (p-S6) is displayed in the aortic endothelium of both WT and *Fbn1*^+/C1039G^ mice. Areas subjected to OSS and NWSS are demarcated by red and yellow dotted outlines, respectively, with nuclei stained by 4′,6-diamidino-2-phenylindole (DAPI). (E, F) Magnified views of p-S6 staining within designated OSS (E) and NWSS (F) regions. (G) Quantitative assessment of p-S6 fluorescence intensity within OSS and NWSS areas demonstrates a significant upregulation of mTORC1 signaling in response to oscillatory shear. Data are mean ± standard error of the mean (SEM) for *n* = 6 mice per group. Statistical analyses were performed using one-way analysis of variance (ANOVA); significance is indicated by ^∗∗∗^*P* < 0.001; NS: not significant.

In *Fbn1*^*+*/C1039G^ mice [25], which model the aortic dilation and aneurysmal pathology characteristic of MFS, hemodynamic assessment revealed no significant differences in systolic or diastolic blood pressure when compared to WT mice. Detailed hemodynamic profiling through color Doppler ultrasound identified pronounced DF in the ascending aorta of *Fbn1*^*+*/C1039G^ mice relative to WT controls, with the former exhibiting flow perturbations particularly in regions of aortic dilation (Fig. 2A, denoted by white arrowheads). To further elucidate the hemodynamic patterns, CFD analyses were employed, recapitulating the blood flow streamlines and velocities within the thoracic aorta. Echoing the ultrasound findings, the CFD simulations exposed substantial DF in the dilated aortic segment of the *Fbn1*^*+*/C1039G^ group, as indicated by red arrowheads (Fig. 2B). The CFD-rendered WSS distribution revealed regions of OSS precipitated by the DF, encompassing the ascending aorta and aortic arch, outlined by red dotted boxes, with red arrowheads pinpointing the OSS regions (Fig. 2C). In contrast, areas subjected to laminar flow with higher velocity exhibited normal wall shear stress (NWSS), colored in gradients from light blue to green (yellow arrowheads and yellow dotted box, Fig. 2C).

Delving into the relationship between these hemodynamic forces and mTORC1 signaling, immunofluorescence imaging of the thoracic aorta highlighted a significant surge in p-S6 expression in the *Fbn1*^*+*/C1039G^ mice within OSS regions (Fig. 2D, red dotted box). In contrast, NWSS areas displayed minimal p-S6 expression (Fig. 2C, yellow dotted box). 3D reconstructions provided a holistic view of p-S6 distribution throughout the aortic media, indicating an upregulation of mTORC1 signaling in the deeper layers of the aortic media of *Fbn1*^*+*/C1039G^ mice (Fig. 2E). Subsequent En face staining confirmed a pronounced enhancement of p-S6 intensity within the OSS domains of the *Fbn1*^*+*/C1039G^ mice, a pattern not observed within NWSS regions in either the *Fbn1*^*+*/C1039G^ or WT mice (Figs. 2F and G). Collectively, these results suggest a direct association between increased mTORC1 pathway activation and the DF dynamics present in the ascending aorta, potentially implicating OSS as a mediator of pathogenic signaling in the context of aortic aneurysm development.

### 3.3 mTORC1 pathway is activated by DF-induced OSS *in vivo*

*Fbn1*^*+*/C1039G^ mice reproduce the pathological changes of aortic dilation and aneurysm found in MFS patients. Systolic and diastolic blood pressure was measured and no significant difference was found between the *Fbn1*^+/C1039G^ and WT mice (Fig. S3). Color Doppler ultrasound was used to assess the hemodynamic characteristics of the aorta. Substantial DF was detected in the ascending aorta of the *Fbn1*^+/C1039G^ mice compared with the WT mice (Fig. 2A, white arrowheads). In particular, the DF overlays the regions of thoracic aortic dilation in *Fbn1*^+/C1039G^ mice. By using CFD analysis, we simulated the streamlines and velocity of the blood flows in the thoracic aorta of the mice. Consistent with the Doppler ultrasound results, abundant DF was observed at the dilated area of the ascending aorta in the *Fbn1*^+/C1039G^ group (red arrowheads in Fig. 2B). The stream lines and the distribution of WSS in the thoracic aortas were rendered. There were OSS regions (marked by the dark blue areas in Fig. 2C) that were induced by the DF, which included the ascending aorta and aortic curvature (indicated by red dotted box and black arrowheads indicated the region of OSS, Fig. 2C). The laminar flows with high velocity triggered NWSS cover the aortic wall with light blue to green areas (indicated by yellow dotted box indicated the region of NWSS, Fig. 2C).

We next investigated the association between the hemodynamic characteristics and mTORC signaling. Immunofluorescence images of the thoracic aorta showed a significantly increased expression of p-S6 in the *Fbn1*^+/C1039G^ mice compared with that of the controls at the regions of OSS and NWSS (red and yellow dotted box, Fig. 2D and E). As a comparison, the areas underlying NWSS (yellow dotted box, Fig. 2F) showed insignificant expression of p-S6. 3D reconstruction images were established to observe the spatial configuration of p-S6 expression in the entire aortic media (Fig. S4). The results showed an elevation of p-S6 signaling in the deep layers of the aortic media in the *Fbn1*^*+*/C1039G^ mice (Fig. S4). En face staining further revealed that the intensity of p-S6 was markedly enhanced in the OSS areas (red dotted boxes) of the *Fbn1*^*+*/C1039G^ mice (Figs. 2E and G). However, the intensity of p-S6 was not significantly increased at the NWSS areas (yellow dotted boxes) in both *Fbn1*^*+*/C1039G^ and WT mice (Figs. 2F and G). These data indicated that increased mTORC1 activation was associated with DF in the ascending aorta.

### 3.4 Elevated mTORC1 activation correlates with progressive aortic dilation and DF dynamics

**Fig. 3:**
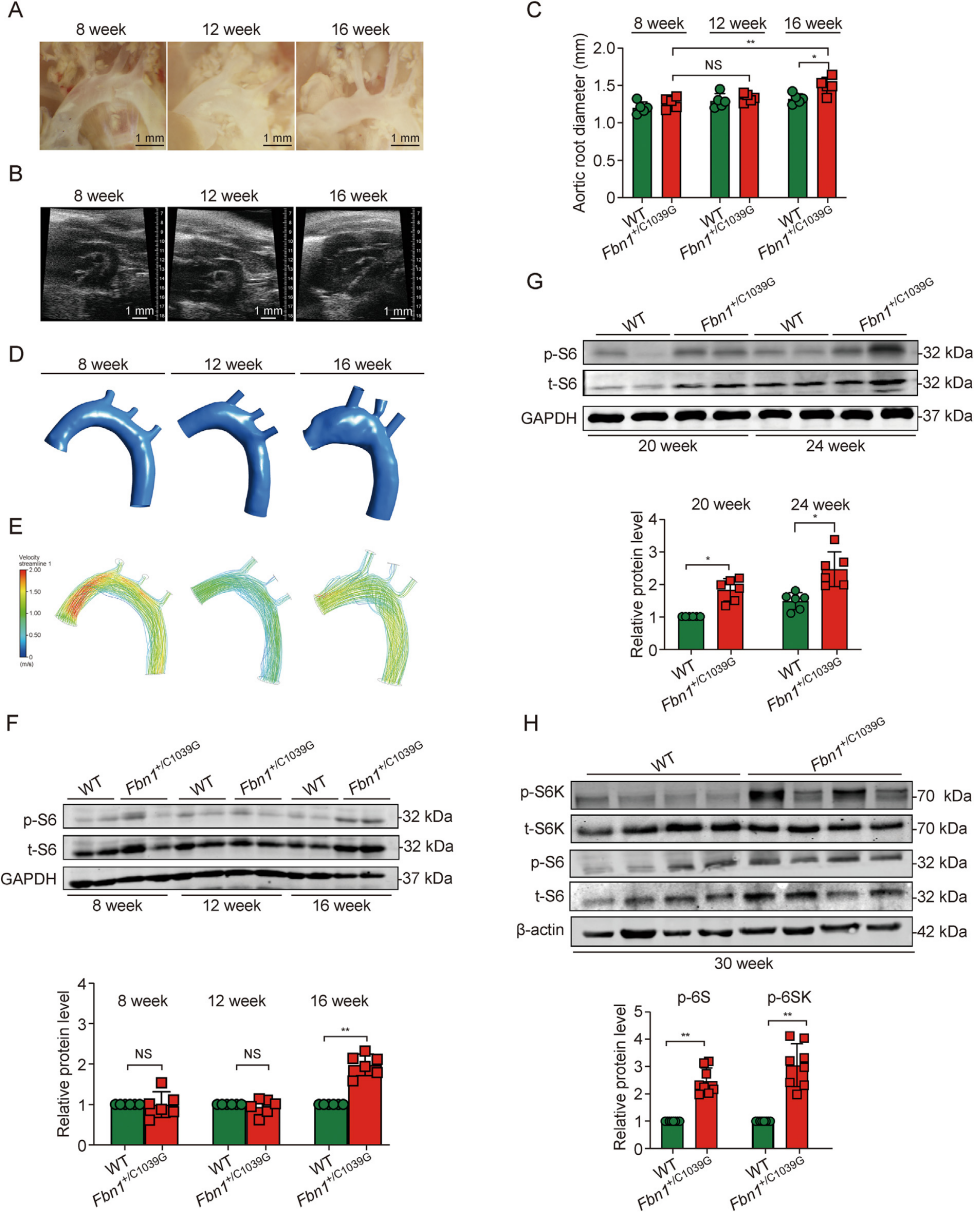
Mechanistic target of rapamycin complex 1 (mTORC1) pathway intensification in *Fbn1*^*+*/C1039G^ mouse aortas over time. (A) Morphology of ascending aortas at different growth stages. (B) Ultrasound showing aortic dilation (end-systolic diameter of the aorta) of *Fbn1*^*+*/C1039G^ mice at 8, 12, and 16 week. (C) The analysis of aortic root diameter tracking by ultrasound of *Fbn1*^*+*/+^ wild-type (WT) and *Fbn1*^*+*/C1039G^ mice. (D) 3D reconstructions of aortic progression over time. (E) Computational fluid dynamic (CFD) analyses indicate increased disturbed flow correlating with aortic expansion. (F) Western blots and the relative protein level of phosphorylated S6 (p-S6), total S6 (t-S6), and glyceraldehyde-3-phosphate dehydrogenase (GAPDH) of the WT and *Fbn1*^*+*/C1039G^ mice at 8, 12, and 16 week. (G) Western blots and the relative protein level of p-S6, t-S6, and GAPDH of the WT, *Fbn1*^*+*/C1039G^ at 20 and 24 week. (H) Western blots and the relative protein level of phosphorylated S6 kinase (p-S6K), t-S6K, p-S6, t-S6, and β-actin of the WT and *Fbn1*^*+*/C1039G^ mice at 30 week. Data are presented as mean ± standard error of the mean (SEM) for *n* = 6 per group. Statistical significance was determined by one-way analysis of variance (ANOVA), with ^∗^*P* < 0.05, and ^∗∗^*P* < 0.01, indicating significant differences; NS: not significant.

Previous literature has indicated that the intensification of DF may be associated with the expansion of aortic aneurysm [13,30,32]. Our investigation delineates the dynamic interplay between mTORC1 pathway intensification and the evolution of aortic aneurysms, underscored by the progressive enlargement and DF patterns within the thoracic aorta of *Fbn1*^*+*/C1039G^ mice. Initially, postnatal examinations revealed no significant dilation within the thoracic aortas of these genetically modified mice. However, a temporal assessment demonstrated a gradual aortic expansion commencing from 16 week of age, contrasting with the consistent morphology observed in WT counterparts (Figs. 3A and B). Differential analyses through ultrasound measurements unveiled an inconsequential variation in aortic root diameters between WT and *Fbn1*^*+*/C1039G^ mice within the 8 to 12-week age bracket.

This paradigm shifted markedly post-16 week, wherein *Fbn1*^*+*/C1039G^ mice exhibited significant aortic root enlargement compared to WT mice (Fig. 3C). 3D CT reconstructions further elucidated the aortic morphological progression over time, providing a vivid depiction of aneurysmal development (Fig. 3D). Complementing structural assessments, CFD analyses illuminated the onset of increasingly DF patterns concurrent with aneurysmal expansion post-12 week, suggesting a correlation between DF dynamics and the aneurysmal enlargement of the aorta (Fig. 3E). This finding posits that the DF may exacerbate the pathological remodeling observed in aneurysmal progression.

Longitudinal analysis of mTORC1 pathway signaling, substantiated through Western blot quantifications, identified a critical inflection point at 16 week. Prior to this milestone, the p-S6 levels in *Fbn1*^*+*/C1039G^ mice were comparable to WT controls, indicative of a pre-dilation phase characterized by stable mTORC1 activity. Beyond this temporal marker, concomitant with the initiation of aortic dilation, we observed a substantial increase in p-S6 levels, intimating a complex relationship between mTORC1(Representative by p-S6) activation and the progression of aortic aneurysms (Fig. 3F). To further investigate whether the augmentation of the mTORC1 pathway is a defining characteristic of TAA development, we conducted a time-extended analysis. This investigation, stratified by the severity of aortic dilation at 20, 24, and 30 weeks, highlighted a marked increase in mTORC1 pathway (Representative by p-S6 and p-S6K) constituents in *Fbn1*^+/C1039G^ mice with pronounced aneurysmal dilation (Figs. 3G and H). Post-16 week, the emergence of TAAs in *Fbn1*^*+*/C1039G^ mice was associated with significantly higher levels of phosphorylated ribosomal p-S6K and p-S6 compared to WT mice, affirming the central role of mTORC1 signaling in the pathogenic enlargement of TAAs (Fig. 3).

In conclusion, our findings assert a direct linkage between the activation of the mTORC1 pathway and the temporal as well as morphological evolution of thoracic aortic aneurysms, further amplified by the interplay with disturbed hemodynamic flow and aortic dilation. This association highlights the therapeutic promise of targeting mTORC1 signaling pathways to potentially impede the progression of TAA in at-risk groups.

### 3.5 Inhibition of mTORC1 pathway ameliorates thoracic aortic aneurysm progression induced by OSS *in vivo*

**Fig. 4:**
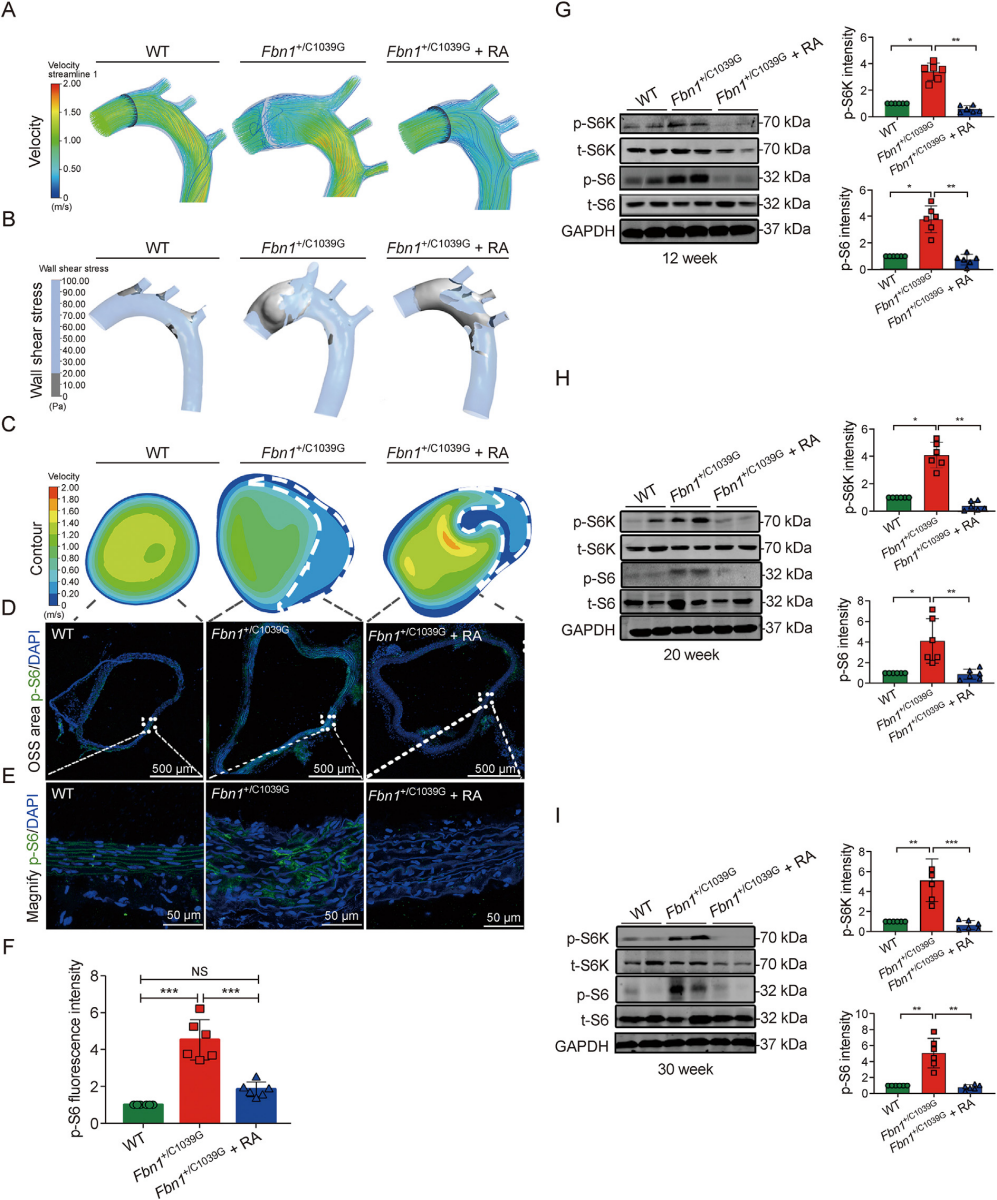
Rapamycin mitigates mechanistic target of rapamycin complex 1 (mTORC1) activation induced by oscillatory shear stress (OSS) in *Fbn1*^+/C1039G^ Mice. (A) Visualization of blood flow velocity and streamlines within the aortic lumen captures the dynamic hemodynamic alterations related to aneurysm progression and the disturbed flow of the three groups: wild-type (WT) littermates, *Fbn1*^+/C1039G^ mice, and *Fbn1*^+/C1039G^ mice subjected to long-term rapamycin treatment (*Fbn1*^+/C1039G^ + RA). (B) The presence of OSS across the aortic regions of the three groups: WT littermates, *Fbn1*^+/C1039G^ mice, and *Fbn1*^+/C1039G^ + RA, with OSS areas marked in gray. (C) Comparative aortic velocity mapping and cross-sectional OSS distribution among the three groups, with OSS zones delineated by white dashed lines. (D) Immunohistochemical detection of phosphorylated S6 (p-S6) in ascending aortic sections from the three groups. (E) Enlarged view of the OSS regions from the respective groups. (F) Quantification of p-S6 fluorescence intensity across the groups, indicating the modulation of mTORC1 signaling by rapamycin. (G–I) Western blot analysis profiling the expression of phosphorylated S6 kinase (p-S6K), total S6 kinase (t-S6K), phosphorylated S6 (p-S6), total S6 (t-S6), and statistical evaluation of protein expressions at 12 (G), 20 (H), and 30 week (I). Data expressed as mean ± standard error of the mean (SEM) for *n* = 6 per group. One-way analysis of variance (ANOVA) was utilized to assess statistical significance, denoted by ^∗^*P* < 0.05, ^∗∗^*P* < 0.01, and ^∗∗∗^*P* < 0.001; NS: not significant.

Our investigation elucidated the interplay between mTORC1 activity and the biomechanical milieu imposed by OSS within the aortic environment *in vivo*. Utilizing CFD analysis, we delineated the OSS profiles within aortic structures, identifying the DF as the primary impetus for OSS genesis, with OSS regions consistently overlapping DF regions (Figs. 4A and B). Our comparative investigations revealed an augmented OSS footprint in the thoracic aortas of *Fbn1*^*+*/C1039G^ mice relative to their WT counterparts, emphasizing the influence of genetic predisposition on hemodynamic stress responses (Figs. 4A and B). Fig. 4B illustrates the distribution of OSS regions (shown in gray) induced by DF in the aortas of three groups: WT mice, *Fbn1*^*+*/C1039G^ mice, and *Fbn1*^*+*/C1039G^ mice with long-term rapamycin treatment (*Fbn1*^*+*/C1039G^ mice + RA). The *Fbn1*^*+*/C1039G^ + RA group received long-term (22 week) rapamycin treatment starting at 8 week and ending at 30 week, covering adolescence to adulthood. We delineated representative cross-sectional planes of the aortas to further analyze hemodynamics on the transverse plane (Fig. 4C). Blood flow in disturbed regions caused local OSS areas around the aortic wall inside the aneurysm sac (encircled with white dashed lines, Fig. 4C) in all three groups.

Using CFD analysis, we employed immunofluorescence to identify p-S6 intensity at OSS regions among the groups (Figs. 4D and E). mTORC1 expression was significantly increased in the VSMCs of *Fbn1*^*+*/C1039G^ mice compared to WT mice. Despite the persistence of OSS, long-term rapamycin treatment inhibited p-S6 activation in the *Fbn1*^*+*/C1039G^ + RA group (Fig. 4F). 3D confocal microscopy provided a detailed visualization of p-S6 distribution throughout the tunica media (Fig. S4). Immunoblot analysis of 12, 20, and 30-week-old *Fbn1*^*+*/C1039G^ mice reconfirmed the inhibitory effect of rapamycin (Figs. 4G–I). The mTORC1 pathway, indicated by p-S6K and p-S6, progressively increased in *Fbn1*^*+*/C1039G^ mice after 12 week of age and was consistently inhibited by rapamycin. These findings suggest that inhibiting mTORC1 signaling can attenuate thoracic aortic aneurysm progression in regions of DF.

### 3.6 Immunofluorescence analysis of eNOS upregulation and oxidative stress in mouse aorta under OSS and the modulatory role of rapamycin

To further elucidate the mechanistic underpinnings of OSS on endothelial and smooth muscle cells, we conducted immunofluorescence staining on mouse aortic sections. We focused on endothelial nitric oxide synthase (eNOS, also known as NOS3, red), CD31 (yellow) and p-S6 (green), to delineate their expression patterns in response to OSS. Immunofluorescence staining revealed a significant upregulation of NOS3 in endothelial cells located in the OSS regions of the aorta in *Fbn1*^*+*/C1039G^, as identified by CD31 staining (Fig. S5A). This upregulation suggests an endothelial response to OSS characterized by increased nitric oxide production. Concurrently, we observed a marked increase in p-S6 expression in the SMCs (Fig. S5B), indicating enhanced mTORC1 activity. Furthermore, to investigate the therapeutic potential of mTORC1 inhibition, we treated mice with rapamycin. This treatment successfully suppressed the OSS-induced upregulation of NOS3 in endothelial cells and p-S6 in SMCs (Figs. S5C–E). Additionally, we conducted immunofluorescence staining on aortic sections under OSS conditions in *Fbn1*^+/C1039G^ mice, with and without rapamycin treatment. DHE staining demonstrated elevated reactive oxygen species (ROS; red) levels across the vascular wall in *Fbn1*^*+*/C1039G^ under OSS regions (Figs. S6A and B). Notably, RA treatment successfully curtailed this upregulation of DHE expression by inhibiting mTORC1 activity (Figs. S6C–E). These findings emphasize the crucial role of the mTORC1 pathway in cellular responses to disturbed hemodynamics and underscore rapamycin's therapeutic potential in modulating these responses.

### 3.7 Rapamycin ameliorates TAA expansion

**Fig. 5:**
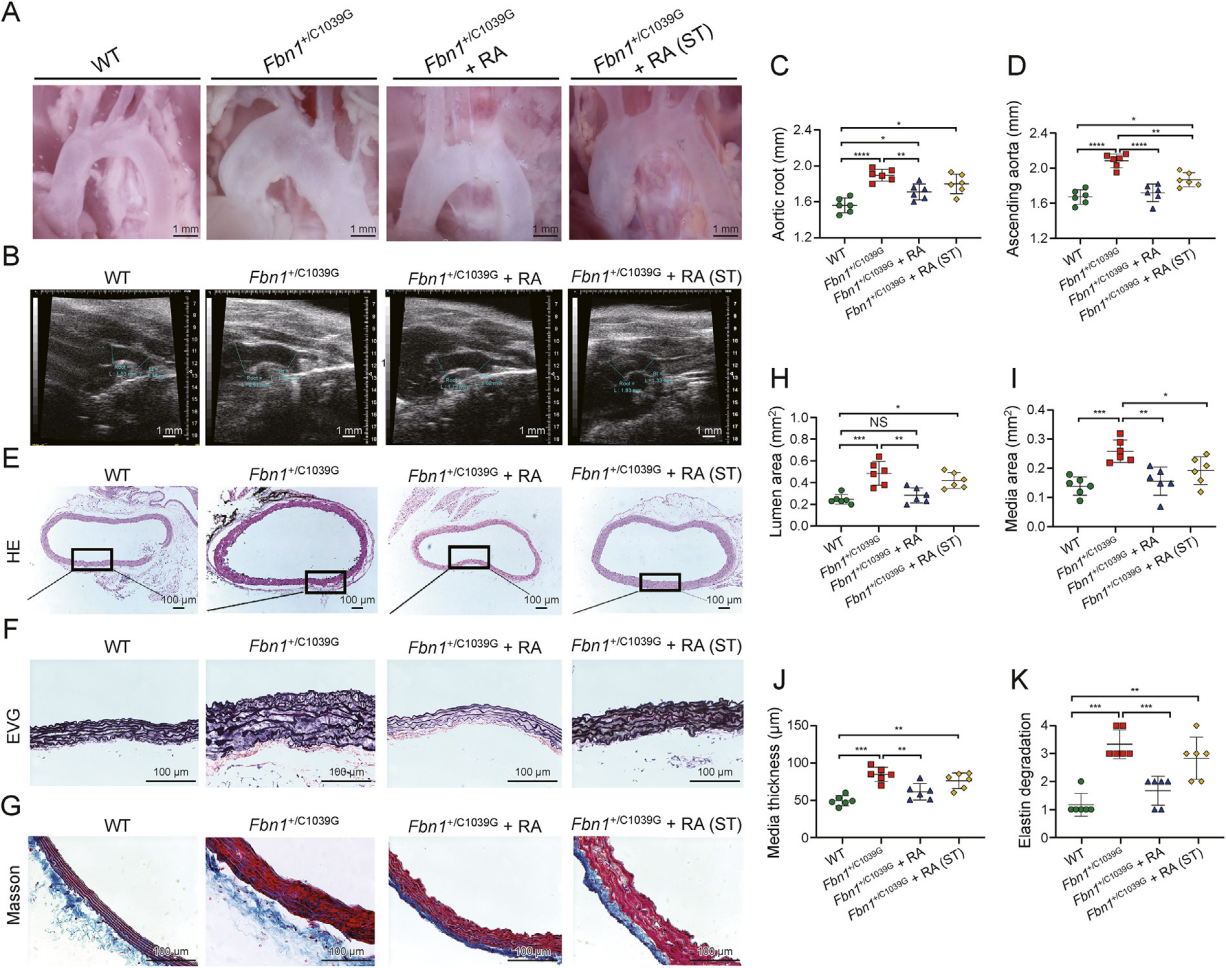
Rapamycin alleviates morphological and structural abnormalities in thoracic aortic aneurysm (TAA) of *Fbn1*^*+*/C1039G^ mice. (A) Ascending aortas from wild-type (WT), *Fbn1*^*+*/C1039G^, and rapamycin-treated *Fbn1*^*+*/C1039G^ mice showcase morphological differences, with treatment durations noted. *Fbn1*^*+*/C1039G^ + RA: *Fbn1*^*+*/C1039G^ mice with long-term rapamycin treatment, starting from 8 and ended at 30 week; *Fbn1*^+/C1039G^ + RA (ST): *Fbn1*^*+*/C1039G^ mice with short-term rapamycin treatment, starting from 12 and ended at 16 week. (B) Representative ultrasound images showing the end-systolic diameter of the ascending aorta and arch from the WT, *Fbn1*^*+*/C1039G^, and *Fbn1*^*+*/C1039G^ mice with long- and short-term rapamycin treatment. (C, D) The measurement of vessel diameters by serial ultrasound on aortic root (C) and ascending aorta (D) at 30 week. (E–G) Hematoxylin and eosin (H&E) (E), elastic van Gieson (EVG) (F), and Masson staining (G) of the aortas collected from 30-week-old mice. (H–K) Statistical analysis of the lumen area (H), media area (I), media thickness (J), and elastin degradation (K) among the four groups. The Mann-Whitney *U* test was used for evaluation of elastin degradation. Data are presented as mean ± standard error of the mean (SEM) for *n* = 6 per group. Statistical significance was determined by one-way analysis of variance (ANOVA), with ^∗^*P* < 0.05, ^∗∗^*P* < 0.01, ^∗∗∗^*P* < 0.001, and ^∗∗∗∗^*P* < 0.0001 indicating significant differences; NS: not significant.

Given rapamycin's potential side effects, our study extended to assess the efficacy of short-term treatment (*Fbn1*^*+*/C1039G^ + RA(ST)) on TAA progression alongside the long-term regimen (*Fbn1*^*+*/C1039G^ + RA). We observed that, while both durations of mTOR inhibition imparted therapeutic benefits, short-term administration of rapamycin partially mitigated the TAA phenotype, albeit to a lesser extent than the long-term treatment (Fig. 5A). Doppler ultrasound assessments revealed that rapamycin significantly reduced the aortic root and ascending aorta dilation observed in *Fbn1*^*+*/C1039G^ mice (Fig. 5B). Importantly, therapeutic effects in curbing aortic enlargement were evident with both treatment timelines, affecting both aortic roots and ascending segments (Figs. 5C and D). Nonetheless, the degree of aortic diameter reduction was less pronounced in the short-term treatment group compared to the long-term regimen (Figs. 5C and D). Histological evaluations confirmed structural improvements in the aorta; rapamycin markedly prevented aortic enlargement, curtailed elastic fiber degradation, and reduced medial thickening (Figs. 5E–K). The treatment effectively restrained the expansion of the medial area, vessel diameter, and lumen area in *Fbn1*^*+*/C1039G^ mice. It is noteworthy that neither systolic nor diastolic blood pressure was altered by the treatment (Fig.S3). Additionally, rapamycin significantly decreased the rate of TAA formation and improved survival rates (Fig. S7). However, a decrease in body weight was observed in the *Fbn1*^*+*/C1039G^ mice post-treatment (Fig. S7).

### 3.8 Therapeutic effects of rapamycin on aortic pathology in *Fbn1*^+/C1039G^ mice

**Fig. 6:**
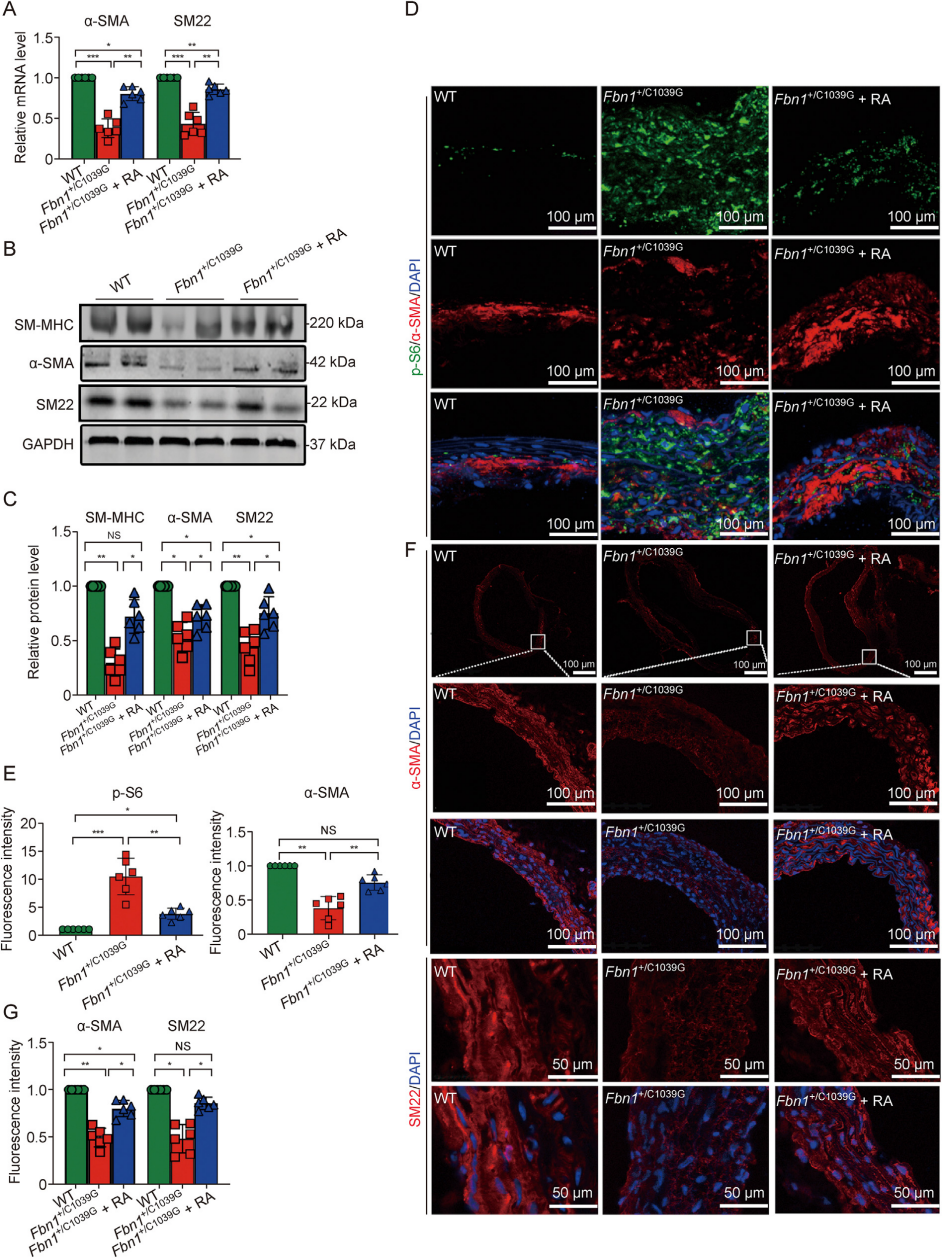
Rapamycin attenuates vascular remodeling in the *Fbn1*^+/C1039G^ Marfan syndrome (MFS) mouse model. (A) mRNA expression of vascular smooth muscle cell (VSMC) markers α-smooth muscle actin (α-SMA) and smooth muscle 22 (SM22) quantified by real-time PCR in ascending aortas of wild-type (WT), *Fbn1*^*+*/C1039G^, and long-term rapamycin-treated *Fbn1*^*+*/C1039G^ + RA mice. (B, C) Western blot (B) and analysis of relative protein levels (C) of smooth muscle myosin heavy chain (SM-MHC), α-SMA, SM22, and glyceraldehyde-3-phosphate dehydrogenase (GAPDH) across groups, with densitometry normalized to WT expression. (D) Immunofluorescence for phosphorylated S6 (p-S6) and α-SMA in aortic sections from each group, visualized with dual labeling (green and red, respectively). (E) Analysis of fluorescence intensity for p-S6 and α-SMA, highlighting the impact of rapamycin on molecular signaling and VSMC phenotype. (F) Further immunofluorescence for α-SMA and SM22, with quantification of fluorescence intensity. (G) Analysis of the fluorscence intensity of α-SMA and SM22. Data are presented as mean ± standard error of the mean (SEM) for *n* = 6 per group. Statistical significance was determined by one-way analysis of variance (ANOVA), with ^∗^*P* < 0.05, ^∗∗^*P* < 0.01 and ^∗∗∗^*P* < 0.001 indicating significant differences; NS: not significant.

In assessing the impact of rapamycin on SMCs, we observed a significant diminution in α-SMA and SM22 RNA expression—markers indicative of the contractile phenotype—in the *Fbn1*^+/C1039G^ group relative to WT. Rapamycin administration elicited an upregulation of these markers, suggesting a restoration of the contractile phenotype (Fig. 6A). Western blot analysis further substantiated these findings, demonstrating that rapamycin treatment ameliorated the decreased expression of key SMC contractile proteins, such as SM-MHC, α-SMA, and SM22 (Figs. 6B and C). Immunofluorescence staining corroborated the Western blot results, revealing enhanced expression of α-SMA and SM22 in rapamycin-treated *Fbn1*^+/C1039G^, indicative of mTORC1 inhibition (Figs. 6 D–G).

**Fig. 7:**
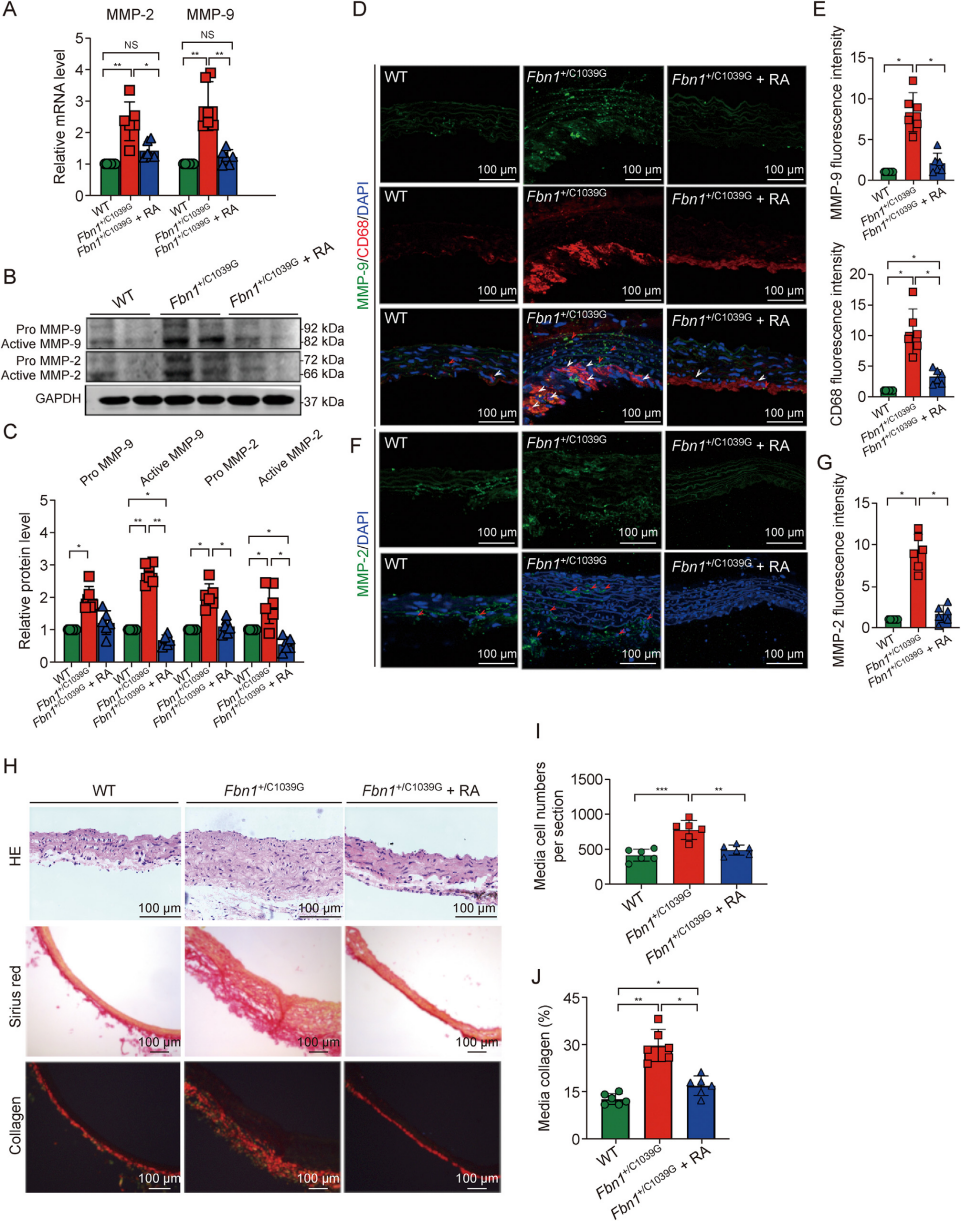
Rapamycin attenuates vascular remodeling by reducing inflammation, oxidative stress, and medial layer hyperplasia. (A) Expression levels of matrix metalloproteinases (MMP-2 and MMP-9) evaluated by real-time PCR, normalized to a reference gene. (B) Western blotting reveals differential expression of pro and active forms of MMP-9 and MMP-2 among the wild-type (WT), *Fbn1*^+/C1039G^, and Fbn1^+/C1039G^ + RA mice. (C) Densitometry of MMPs, with relative band intensities compared to WT controls. (D–G) Immunofluorescent staining and fluorescence intensity for MMP-9, CD68 (D, E) and MMP-2 (F, G) in aortic sections. (H) Hematoxylin and eosin (H&E) and Sirius red staining of aortic sections, demonstrating decreased medial thickening and collagen deposition after rapamycin therapy. (I, J) Medial cellularity (I) and collagen content (J) quantified from stained sections, indicating reduced smooth muscle cell hyperplasia and collagen overaccumulation following treatment. Data are presented as mean ± standard error of the mean (SEM) for *n* = 6 per group. Statistical significance was determined by one-way analysis of variance (ANOVA), with ^∗^*P* < 0.05, ^∗∗^*P* < 0.01 and ^∗∗∗^*P* < 0.001 indicating significant differences; NS: not significant.

**Fig. 8:**
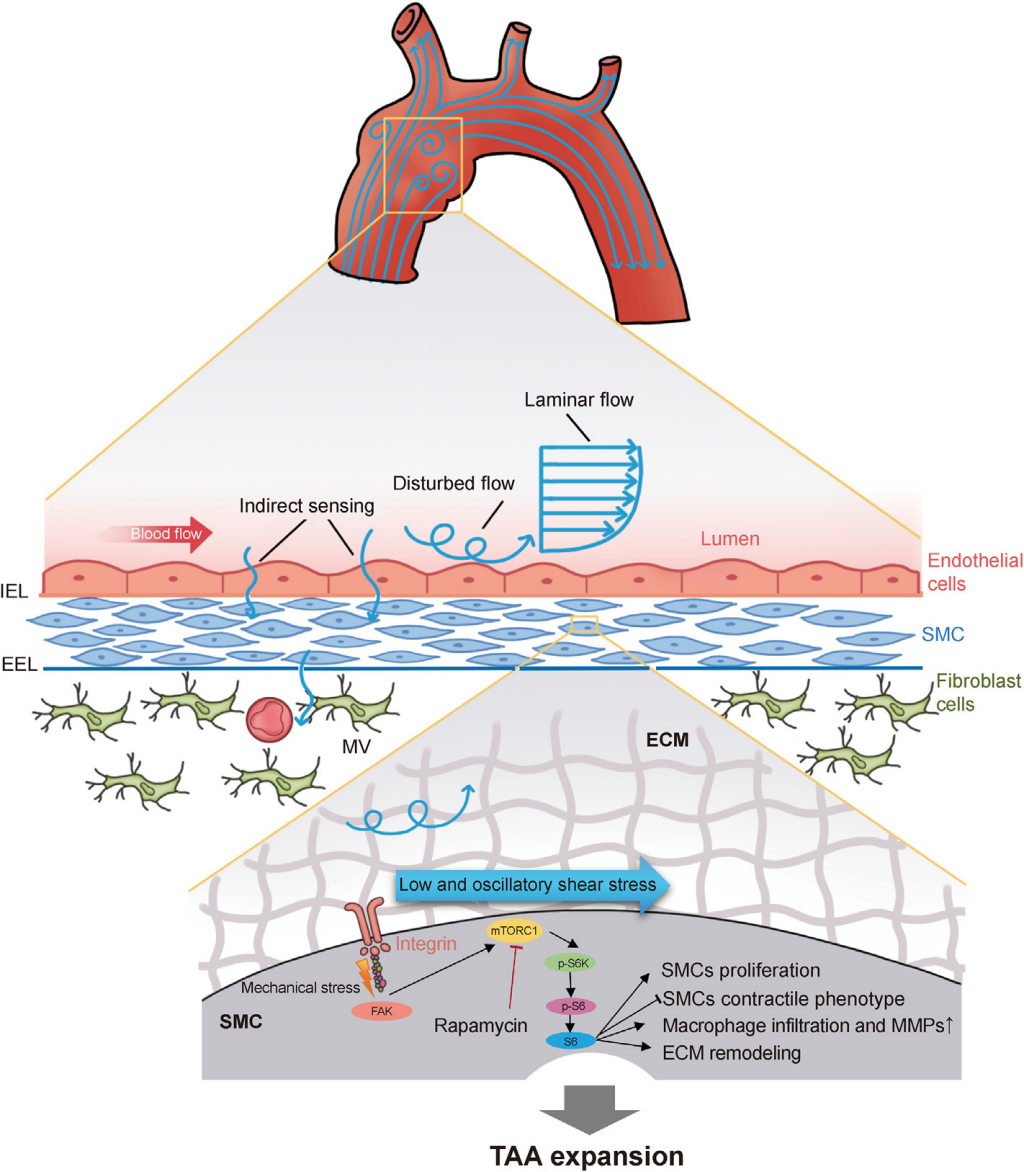
Schematic diagram of mechanistic target of rapamycin complex 1 (mTORC1) pathway-mediated thoracic aortic aneurysm expansion under oscillatory shear stress (OSS) in Marfan syndrome. TAA: thoracic aortic aneurysm; SMC: smooth muscle cell; ECM: extracellular matrix; MVs: micro vessels; MMPs: matrix metalloproteinases; FAK: focal adhesion kinase.

Furthermore, our results delineate rapamycin's efficacy in reducing macrophage infiltration and the expression of matrix metalloproteinases (MMPs) within the aortic media. Notably, MMP-9 and MMP-2, which are implicated in the pathogenesis of TAA) in MFS [33], were significantly elevated in *Fbn1*^+/C1039G^ mice. Rapamycin treatment effectively lowered MMP-9 and MMP-2 levels, both at the mRNA level (Fig. 7A) and protein level (Figs. 7B and C). Immunofluorescence imaging illustrated the overexpression of these MMPs in the medial layer of untreated *Fbn1*^+/C1039G^ mice, which was significantly reduced following rapamycin intervention, indicating attenuation of the inflammatory phenotype (Figs. 7D–G). CD68 expression, a macrophage marker, also diminished with rapamycin treatment (Figs. 7D and E). We quantified the medial SMC proliferation and noted marked hyperplasia in the *Fbn1*^+/C1039G^ group (Fig. 7H) when compared with WT mice. Rapamycin treatment significantly subdued this hyperplastic response (Fig. 7H). Sirius red staining, corroborated by polarized light observation, confirmed that rapamycin mitigated collagen accumulation, underscoring its potential to modulate pathological vascular remodeling (Figs. 7I and J). This therapeutic response to rapamycin correlates with our microarray data from MFS patient samples. These data indicate an mTORC1 signaling hyperactivation in response to DF, which affects VSMC behavior at both genetic and protein levels, as shown in Fig. S8. Specifically, we observed an upsurge in collagen accumulation and cell proliferation, an increase in the synthetic phenotype and degradative activity of VSMCs, alongside a downregulation in elastin synthesis and expression of SMC contractile markers (Fig. S8). Collectively, these findings implicate the mTORC1 pathway inhibition by rapamycin as a key modulator of SMC proliferation, phenotypic stability, macrophage infiltration and extracellular matrix composition (Fig. 8).

## 4 Discussion

In the exploration of TAA pathogenesis within MFS, this study extends beyond conventional pathological frameworks to examine the biomechanical aspects of disease progression. The findings suggest a potential role of DF in exacerbating disease severity, possibly mediated through the activation of the mTORC1 pathway in response to OSS. This observation aligns with previous hypotheses of dysfunctional mechanosensing in aneurysms [6,7,34,35], and may offer insights into a clinical context, contributing to the understanding of the mechanoresponsive nature of the mTORC1 pathway in MFS [[19], [20], [21],^36^].

Our investigation into the mTOR pathway, informed by our prior work [28,31], has now elucidated its potential function in aortic disease models. The question of why mTOR pathway hyperactivation occurs and how its inhibition presents therapeutic benefits has led us to identify the PI3K-Akt-mTOR axis and focal adhesion kinase (FAK) as key mechanotransducers [19]. OSS has been implicated in the sustained activation of the PI3K-Akt-mTOR-p70S6K axis *in vitro* and is a critical risk factor for the dilation and rupture of aortic aneurysms according to biomechanical studies in humans [15,23]. However, corroborative support from *in vivo* studies, whether from animal models or human data, has been lacking. This gap in evidence can be attributed to the absence of precise methods for localizing OSS or NWSS spatial positions *in vivo*, which has hindered accurate assessment of changes in relevant areas [[37], [38], [39]]. Our study addresses this limitation by employing computational fluid dynamics to precisely identify OSS spatial locations. Through comparative analysis with control regions, we observed significantly elevated mTORC1 levels in areas of OSS induced by DF compared to other regions. Importantly, these areas of increased mTORC1 activity spatially corresponded with aneurysm expansion sites. This spatial correlation suggests that mTORC1 may serve as a critical factor in aortic dilation and rupture, bridging the gap between biomechanical stressors and clinical evidence. This insight is valuable, not only for understanding the disease mechanism but also for identifying therapeutic targets. The *in vivo* validation of mTORC1 activation under DF conditions over time reinforces the concept that biomechanical forces may play a crucial role in TAA development in MFS [11,19,36,40], opening avenues for targeted therapeutic interventions.

It is important to note that ECs, being in direct contact with blood flow, are the most sensitive to changes in blood flow dynamics, making them a significant initiating factor in pathological processes. Recent studies have highlighted the critical role of endothelial dysfunction in MFS [41,42], demonstrating that impaired endothelial function correlates with aortic dilation, and that endothelial-specific angiotensin II receptor type I (ATR1) signaling significantly contributes to thoracic aortic aneurysm development, while NOS3 activation may offer therapeutic benefits in preventing disease prone to atherosclerosis [43]. Mieremet et al. [14] investigates OSS and ECs dysfunction in a mouse model of MFS and reveals a crucial role of ECs in MFS aortic pathology and suggest potential new therapeutic targets for MFS treatment. This understanding highlights a potential novel view that endothelial dysfunction caused by OSS subsequently leads to ECM remodeling, which in turn induces changes in SMCs [34].

The present findings showing that OSS augments mTORC1 pathway activation in MFS models. The upregulation of NOS3 and p-S6 in response to OSS further corroborates the hypothesis that OSS-induced endothelial dysfunction and subsequent SMC phenotypic modulation are critical events in TAA pathogenesis. This may be due to OSS-induced upregulation of NOS3 expression, leading to excessive release of nitric oxide from endothelial cells [44], which triggers a feedback regulation in the SMC layer, subsequently resulting in mTORC1 upregulation and potentially increased oxidative stress [45] across the entire vascular wall. Our results provide compelling evidence that OSS-induced upregulation of NOS3 in endothelial cells and p-S6 in VSMCs contributes to the pathophysiology of TAA. Rapamycin's efficacy in reversing these changes highlights the therapeutic potential of targeting the mTORC1 pathway to mitigate TAA progression in MFS. Howerver, further research is needed to fully understand the crosstalk and mutual regulation between ECs and SMCs. The complexity of ECs-to-SMCs interactions underscores the need for more detailed studies to elucidate these mechanisms. This cascade of events highlights the interconnected roles of hemodynamics, endothelial function, and vascular wall remodeling in the pathogenesis of aortic aneurysms, underscoring the complex interplay between mechanical forces and cellular responses in vascular disease progression.

The core pathology in aortic aneurysm disease lies in the aortic media. SMCs, being the primary cell type in the aortic media layer, occupy a central position in aortic aneurysm research [15,21,26]. Therefore, this study focuses on investigating the response of SMCs to biomechanical changes. Our longitudinal analysis suggests that mTORC1 activation, potentially influenced by OSS, may be a dynamic process that correlates with TAA progression. This observed relationship between aneurysm development, altered hemodynamics, and mTORC1 pathway activity warrants further investigation into mTORC1's potential as an indicator of ongoing aortic dilation [18,19,21]. Importantly, our findings indicate that cardiovascular abnormalities in MFS might develop gradually in response to OSS, rather than being solely congenital. This perspective aligns with and extends insights from human MFS studies [9,10], potentially informing future diagnostic and therapeutic strategies. However, we acknowledge that additional research is necessary to fully elucidate these complex relationships and their clinical implications. The study describes a temporal pattern of mTORC1 pathway activation, seemingly dormant postnatally but intensifying concurrent with aortic enlargement, potentially indicating a window for therapeutic intervention around 12 week of age in the mouse model. Two rapamycin treatment regimens were assessed: a long-term protocol initiated at 8 week, which appeared to slow aneurysm progression and enhance survival but raised concerns about weight reduction [21]; and a short-term regimen commenced at 12 week, yielding milder TAA suppression without adverse weight impact. These observations suggest potential avenues for managing aortic expansion in MFS, while highlighting the need for careful consideration of treatment timing and duration. Although the mTOR pathway has been previously reported, the experimental treatment of aortic diseases with rapamycin, much like the discovery of potential benefits of the traditional medicine andrographolide in atherosclerotic cardiovascular diseases [43], represents a case of novel applications for established compounds. The inhibition of mTORC1 by rapamycin might be considered “Novel wine in an old bottle” for the treatment of TAA in MFS. Since previous treatments based on genetic abnormalities have shown limited effectiveness in MFS, additional pharmaceutical research is warranted to investigate strategies from a biomechanical perspective.

Despite these findings, several limitations are acknowledged. The indirect mechanosensing by SMCs, due to their lack of direct exposure to blood flow, and the absence of fluid-structure interaction models in the computational fluid dynamics analysis, indicates areas for future refinement. Moreover, the absence of a comprehensive investigation into the cross-talk between endothelial cells and SMCs leaves unanswered questions about the transmission of endothelial stimuli to the SMCs, emphasizing the need for further research to establish causal relationships and elucidate the precise mechanisms involved in TAA development in MFS. Another limitation of this study is the exclusion of female mice, which was done to control for sex-related confounding variables; however, this approach may limit the generalizability of the findings to both sexes.

## 5 Conclusions

In conclusion, our study not only elucidates the biomechanical role of the mTORC1 pathway in TAA progression within MFS but also positions rapamycin as a promising therapeutic agent. While our findings suggest rapamycin's therapeutic promise, they also underscore the necessity for further research. Future endeavors should focus on the intricate relationship between biomechanical forces and their molecular responses, striving to develop nuanced interventions for TAA.

## CRediT authorship contribution statement

**Ming-Yuan Liu:** Writing – review & editing, Writing – original draft, Visualization, Validation, Funding acquisition, Data curation, Conceptualization. **Meili Wang:** Data curation, Conceptualization. **Junjun Liu:** Formal analysis. **An-Qiang Sun:** Visualization, Conceptualization. **Chang-Shun He:** Formal analysis, Conceptualization. **Xin Cong:** Software, Resources, Methodology. **Wei Kong:** Project administration, Methodology, Investigation. **Wei Li:** Supervision, Project administration, Methodology, Investigation, Conceptualization.

## Data and materials availability

All data needed to evaluate the conclusions in the paper are present in the paper and/or the Supplementary Materials. Additional data related to this paper may be requested from the authors.

## Declaration of competing interest

The authors declare that there are no conflicts of interest.
