# Supplementary material for: Hemodynamic disturbance and mTORC1 activation: Unveiling the biomechanical pathogenesis of thoracic aortic aneurysms in Marfan syndrome
Source: J Pharm Anal. 2024 Oct 28;15(2):101120. doi: 10.1016/j.jpha.2024.101120 (PMC11847113; doi:10.1016/j.jpha.2024.101120)
Supplement: Multimedia component 1 [file mmc1.docx]

Supplemental materials and the legends

**Figs. S1-8**


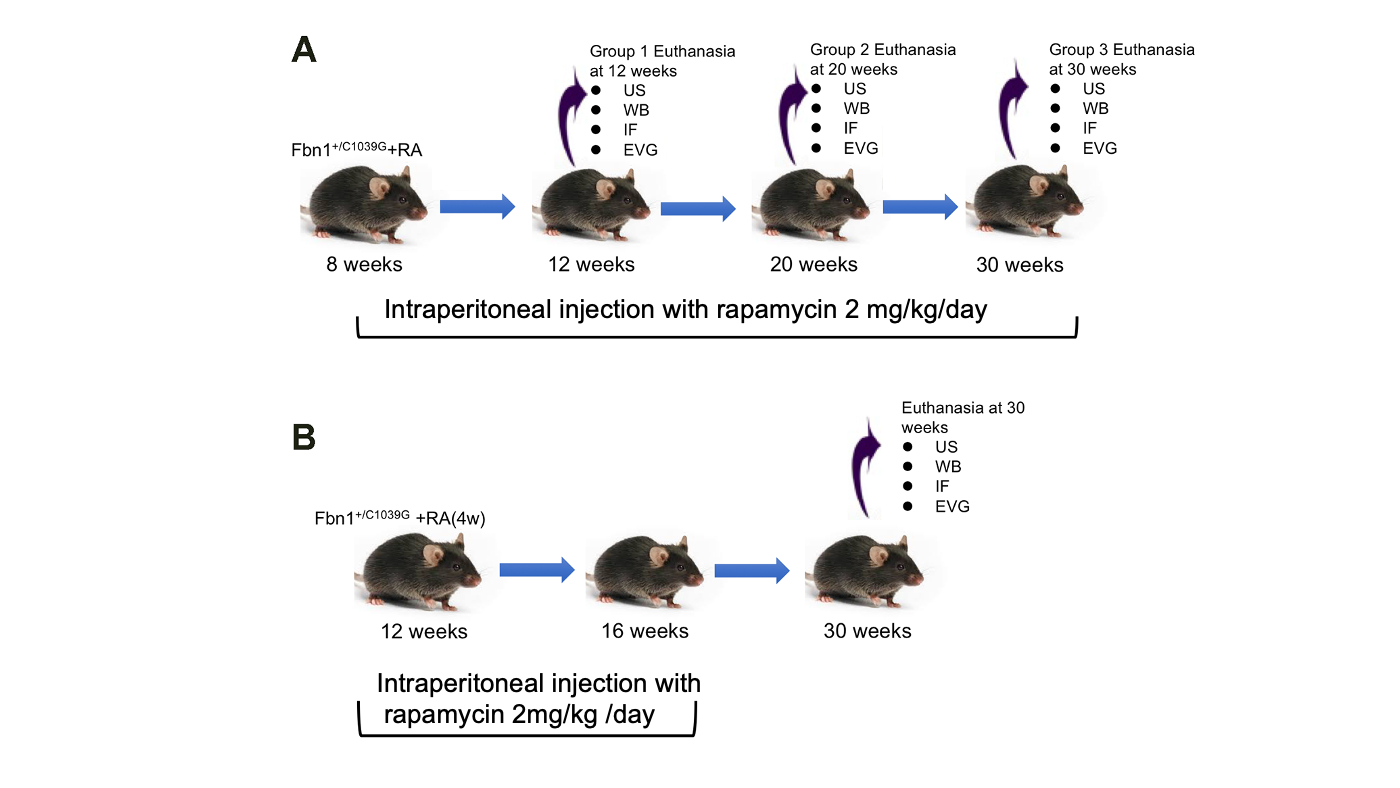


**Supplemental Figs. 1. The diagram of rapamycin treatment in fibrillin-1 (Fbn1) ^+/C1039G^ mice.**

The Flow diagram of rapamycin administration for long-term (22 weeks) treatment (A) Fbn1^+/C1039G^+RA and short-term (4 weeks) treatment (B) Fbn1^+/C1039G^+RA (ST). Fbn1^+/C1039G^+RA indicated Fbn1^+/C1039G^ mice treated with rapamycin（2mg/kg, i.p. q.d.） from 8 to 30 weeks, which represented the long-term treatment model that have covered the adolescent to adulthood; Fbn1^+/C1039G^+RA (4w) indicated Fbn1^+/C1039G^ mice treated with rapamycin（2mg/kg, i.p. q.d.）from 12 to 16 weeks, which represented the short-term treatment model after the adulthood. RA, rapamycin. US, ultrasound. WB, Western blot. IF, immunofluorescence. EVG, Elastic *Van Gieson*.


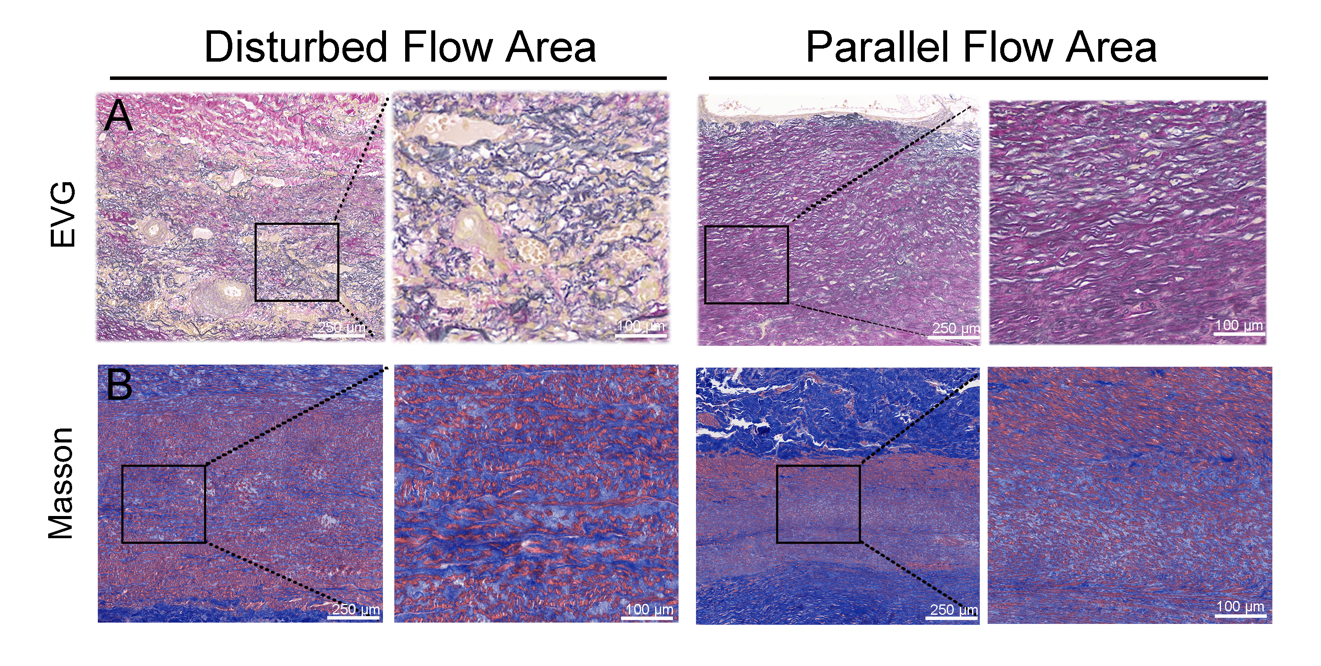


**Supplemental Figs. 2 Elastic *Van Gieson* (EVG), Masson staining, Immunohistochemistry staining of phosphorylated** **extracellular signal-regulated kinase (p-ERK) and phosphorylated Smad2 (p-Smad2) in the disturbed and parallel flow areas of the aneurysm aorta from MFS patients.**

EVG (A) and Masson (B) stains of the abdominal aortic aneurysm of a MFS patient demonstrating the elastin degradation, media thickening and collagen deposition of the disturbed flow area and parallel flow area, black bar=250 μm; white bar=100 μm.

**
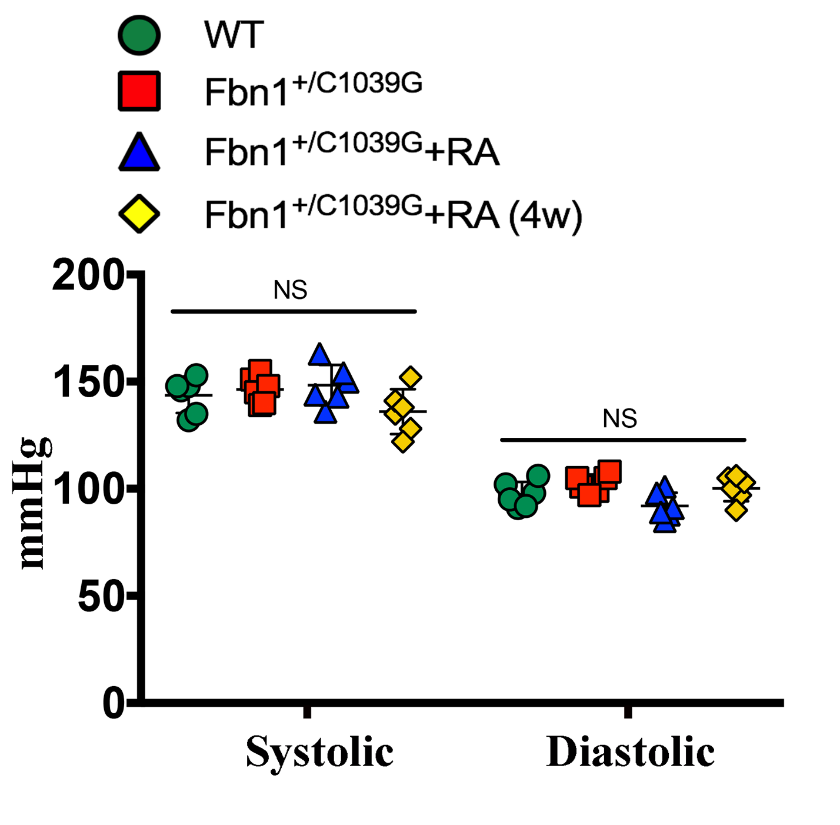
**

**Supplemental Figs. 3. The systolic and diastolic pressure in fibrillin-1 (Fbn1) ^+/C1039G^ mice.** The blood pressure was detected by tail-cuff method in 30-week-old Fbn1^+/+^ wildtype (WT) littermates, Fbn1^+/C1039G^ mice, and Fbn1^+/C1039G^ mice with rapamycin (RA) treatment for long-term (22 w, starting from 8 w to 30 w) and short-term (4 w, starting from 12 w to 16 w). The data were shown as mean ± SEM, and detected by one-way ANOVA. N = 6 per group. NS, no significance.


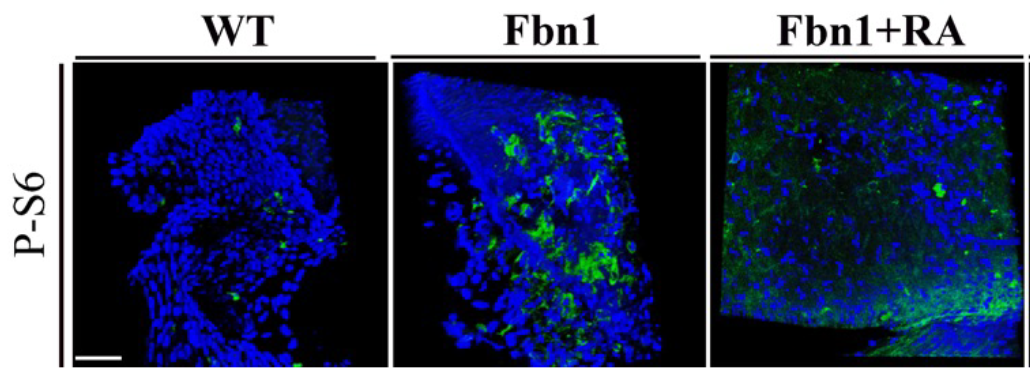


**Supplemental Figs. 4. The mTORC1 activation was observed in the entire tunica media.**

3D confocal laser-scanning microscopy detection for the expression of Phosphorylate S6 (green) in the entire media layer of the respective mice aorta, bar=50 μm; WT, wild type; Fbn, Fbn1^+/C1039G^; Fbn1^+/C1039G^+RA, Fbn1^+/C1039G^ mice with rapamycin treatment from 8 weeks to euthanasia.


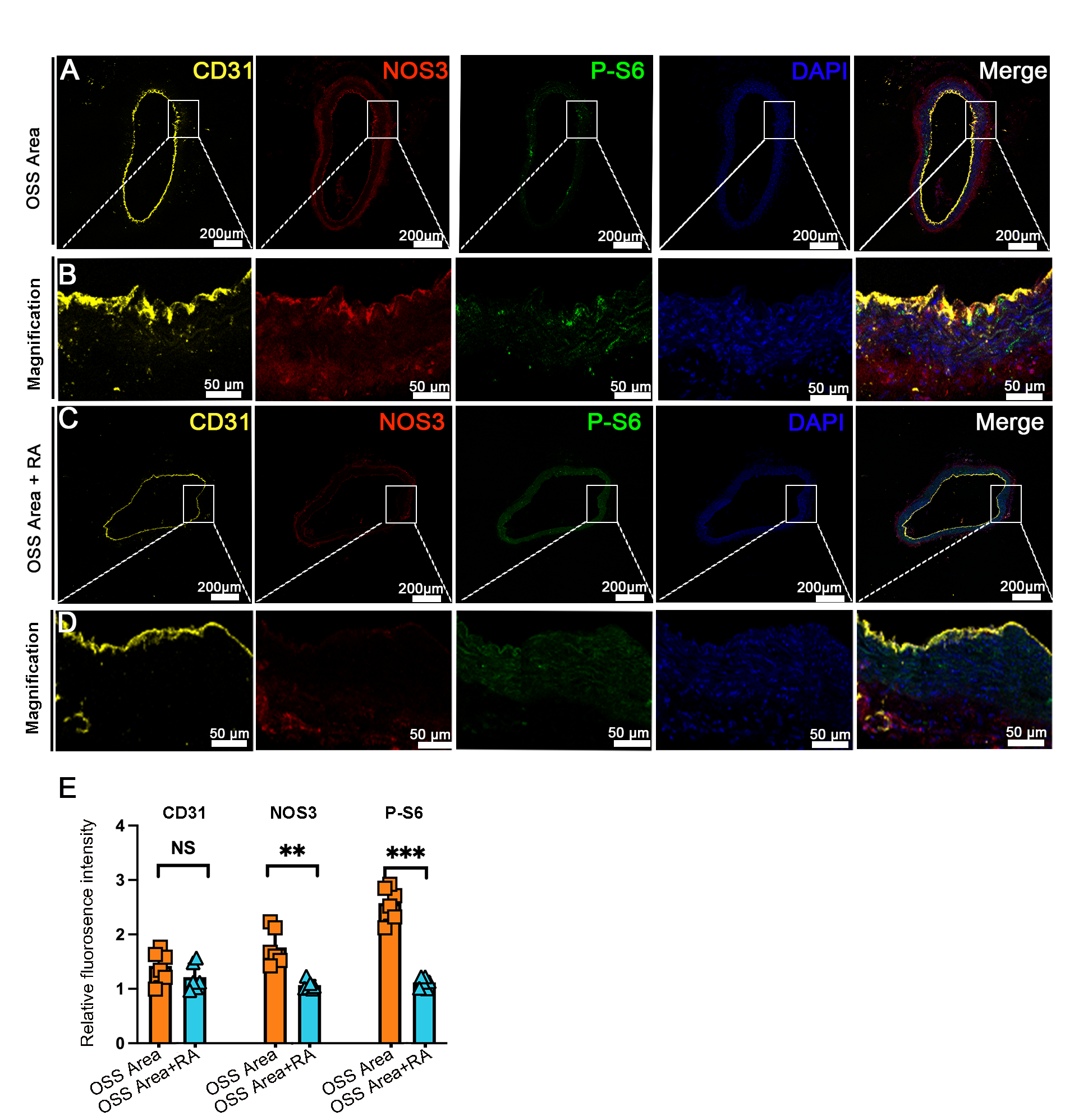


**Supplemental Figs. 5. Immunofluorescence analysis of eNOS (NOS3) and P-S6 expression in Disturbed Flow (DF) Regions of the Aorta**

Immunofluorescence staining of aortic regions under oscillatory shear stress (OSS) from Fbn1^+/C1039G^ and Fbn1^+/C1039G^mice treated with rapamycin (RA). Sections were stained for endothelial cell marker CD31 (yellow), endothelial nitric oxide synthase (NOS3) (red), phosphorylated ribosomal protein S6 (P-S6) (green), and nuclei (DAPI, blue). The merged images combine all four stainings. (A) Low magnification images of aortic sections from untreated Fbn1^+/C1039G^, highlighting the localization of CD31, NOS3, P-S6, and DAPI. (B) Higher magnification of the boxed area in (A) showing detailed localization of CD31, NOS3, and P-S6 in the endothelium and smooth muscle cells. (C) Low magnification images of aortic sections from Fbn1^+/C1039G^+RA mice, showing the effect of rapamycin on the expression of CD31, NOS3, P-S6, and DAPI. (D) Higher magnification of the boxed area in (C), detailing the effect of RA treatment on the expression of CD31, NOS3, and P-S6. (E) Quantification of relative protein levels of CD31, NOS3, and P-S6 in aortic sections from untreated (red) and RA-treated (blue) Fbn1^+/C1039G^ mice. Data are presented as mean ± SEM (n=6 per group). Statistical significance was determined by one-way ANOVA: NS, not significant; **P < 0.01; ***P < 0.001.


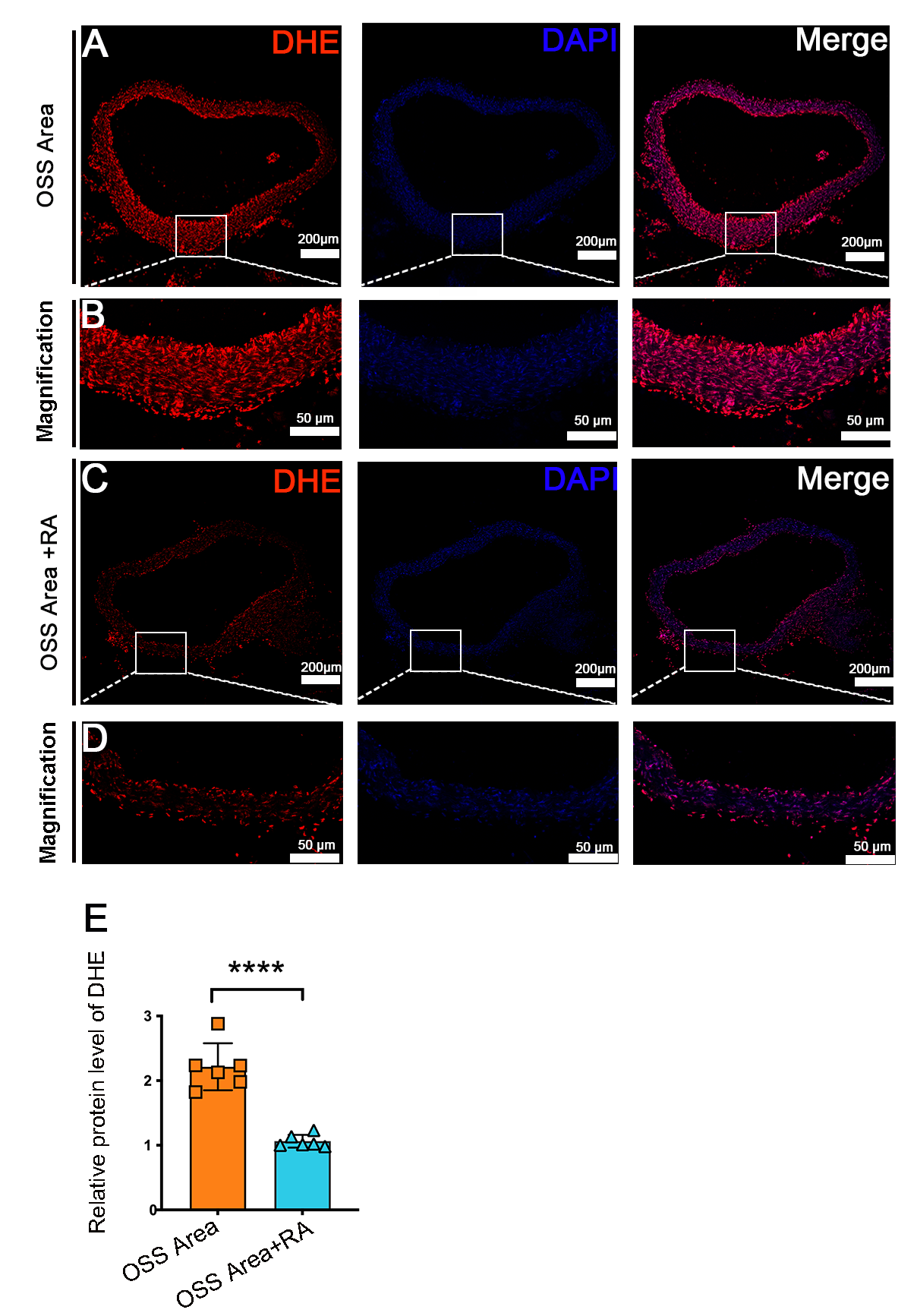


**Supplemental Figs. 6. Immunofluorescence Analysis of Oxidative Stress in Disturbed Flow (DF) Regions of the Aorta**

Immunofluorescence staining of aortic regions under oscillatory shear stress (OSS) from from Fbn1^+/C1039G^ and Fbn1^+/C1039G^ mice treated with rapamycin (RA). Sections were stained for dihydroethidium (DHE, red) to detect reactive oxygen species (ROS) and DAPI (blue) to stain nuclei. The merged images combine both stainings. **(A)** Low magnification images of aortic sections from untreated Fbn1^+/C1039G^ mice, highlighting the distribution of ROS (DHE staining) and nuclei (DAPI staining).**(B)** Higher magnification of the boxed area in (A) showing detailed localization of ROS in the vascular wall.**(C)** Low magnification images of aortic sections from Fbn1^+/C1039G^ mice treated with RA, showing the effect of rapamycin on ROS levels and nuclei.**(D)** Higher magnification of the boxed area in (C), detailing the effect of RA treatment on ROS levels in the vascular wall. **(E)** Quantification of relative protein levels of ROS (DHE fluorescence intensity) in aortic sections from untreated (red) and RA-treated (blue) Fbn1^+/C1039G^ mice. Data are presented as mean ± SEM (n=6 per group). Statistical significance was determined by one-way ANOVA: ****P < 0.0001.


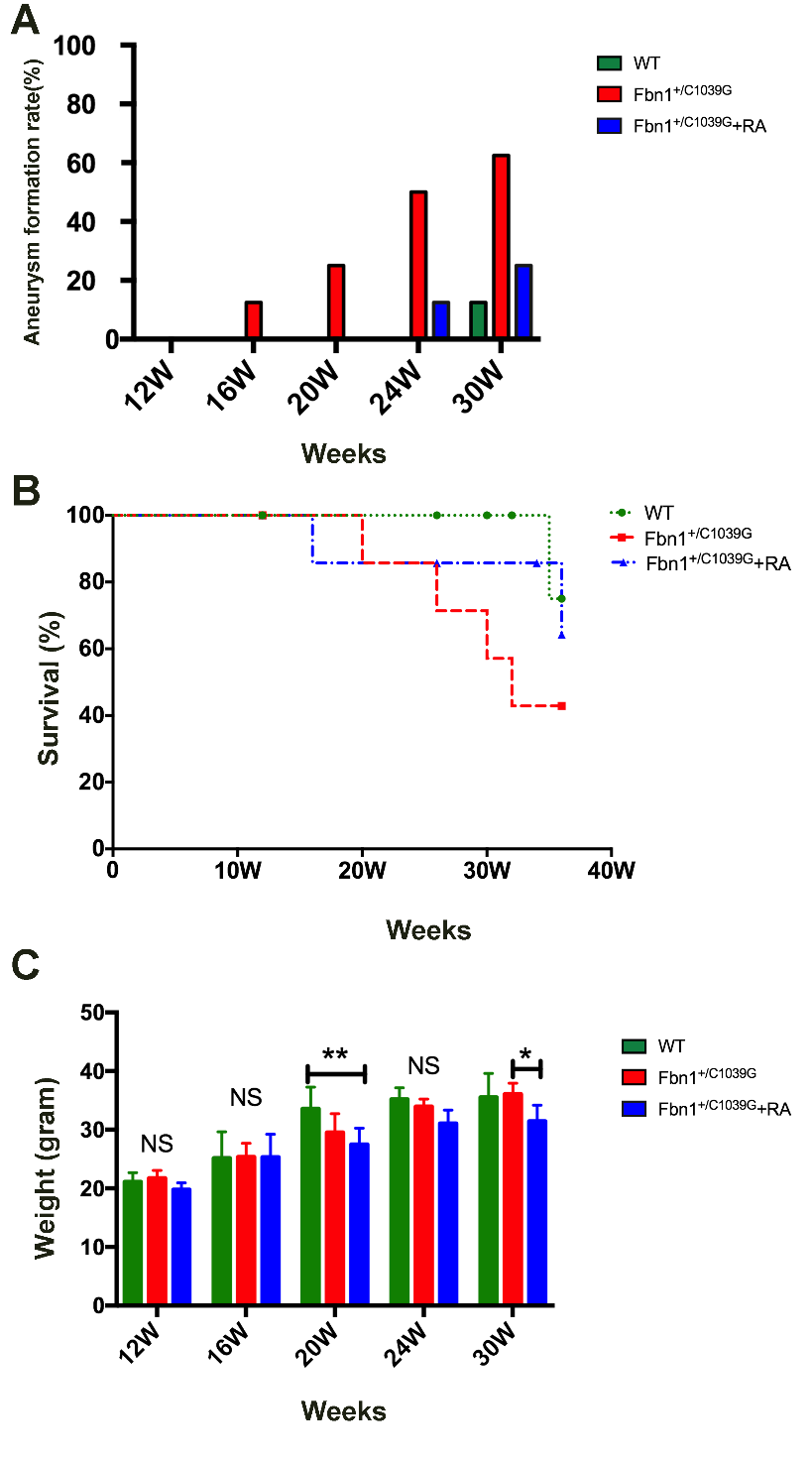


**Supplemental Figs. 7. The effects of rapamycin administration on aneurysmal formation rate, survival, and body weight in fibrillin-1 (Fbn1) ^+/C1039G^ mice.**

(A) The percentage of thoracic aortic aneurysm formation of Fbn1^+/+^ wildtype (WT) littermates, Fbn1^+/C1039G^ mice, and Fbn1^+/C1039G^ mice with rapamycin (RA) treatment over time. (B) The Kaplan-Meier survival curve of WT, Fbn1^+/C1039G^, and Fbn1^+/C1039G^ +RA mice. (C) The changes of body weight over time after receiving rapamycin treatment. The data were shown as mean ± SEM. N = 6 per group. ** *P* < 0.01 and * *P* < 0.05 by two-way ANOVA. NS, no significance.

**Supplemental Figs. 8. Differential Gene and Protein Expression in VSMCs Under Disturbed Versus Laminar Flow in Marfan Syndrome**
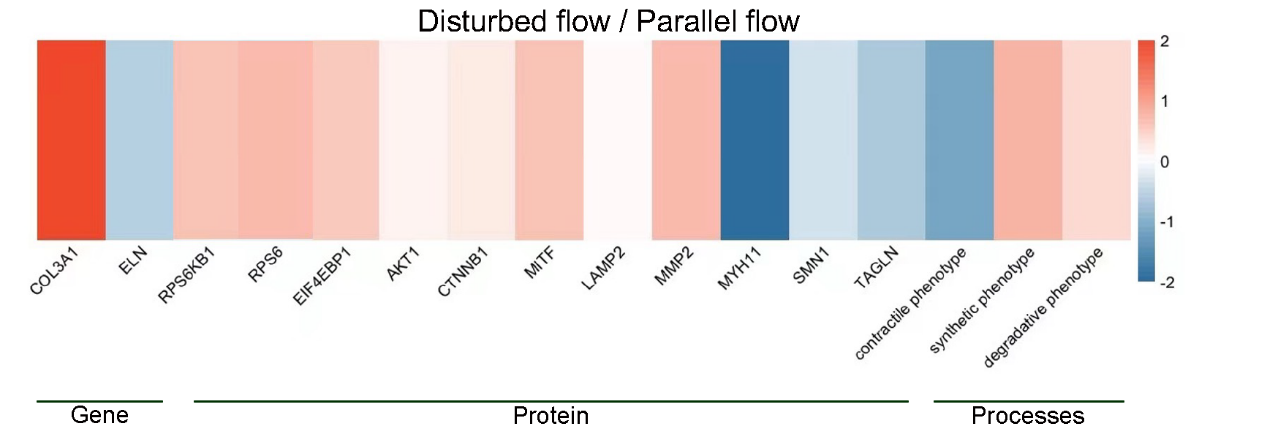


Our RNA-sequencing analysis delineates the differential expression profiles in vascular smooth muscle cells (VSMCs) from MFS patients under disturbed flow compared to parallel flow conditions. The data reveal a pronounced hyperactivation of mTORC1 signaling pathways, triggering both upregulation and downregulation of various signaling entities at the gene and protein levels. This qualitative signaling network model encapsulates the observed experimental variations, illustrating an increased activity in collagen synthesis, cell proliferation, synthetic phenotype markers, and proteolytic enzymes, contrasted by a downregulation of elastin and SMC-specific contractile proteins. Specifically, entities downstream of mTORC1, such as p-S6K, p-S6, and p-4EBP1, manifested a marked increase in the regions of disturbed flow. Conversely, upstream components like p-AKT showed a moderate elevation. Additionally, β-catenin, cadherin-associated protein beta 1 (CTNNB1), microphthalmia-associated transcription factor (MITF), and the proteolytic enzyme matrix metalloproteinase-2 (MMP2) were upregulated under disturbed flow conditions. Contractile protein levels, including smooth muscle myosin heavy chain (SMMHC), α-smooth muscle actin (SMA), and SM22, exhibited a decline. In terms of extracellular matrix (ECM) composition, there was a notable increase in the alpha 1 chain of type III collagen (COL3A1), whereas elastin (Eln) gene expression was diminished under disturbed flow.

**Table S1. List of primers for quantitative reverse-transcription PCR (qRT-PCR)**

| Target | Forward primer (5’-3’) | Reverse primer (5’-3’) |
| --- | --- | --- |
| Mouse α-SMA | CCCAGACATCAGGGAGTAATGG | TCTATCGGTACTTCAGCGTCA |
| Mouse SM22 | CCAACAAGGGTCCATCCTAC | ATCTGGGCGGCCTACATCA |
| Mouse MMP-2 | GACCAGAACACCATCGAGAC | GTGACGTCGCTCCATACTTT |
| Mouse MMP-9 | GTGACGTCGCTCCATACTTT | AAGGTTTGGAATCGACCCAC |
| Mouse GAPDH | AATGGATTTGGACGCATTGGT | TTTGCACTGGTACGTGTTGAT |
