# Supplementary material for: Hemodynamic disturbance and mTORC1 activation: Unveiling the biomechanical pathogenesis of thoracic aortic aneurysms in Marfan syndrome
Source: J Pharm Anal. 2024 Oct 28;15(2):101120. doi: 10.1016/j.jpha.2024.101120 (PMC11847113; doi:10.1016/j.jpha.2024.101120)
Supplement: Multimedia component 2 [file mmc2.pdf]

北京大学人民医院医学伦理委员会
伦理审查意见

意 见 号	2017PHB166-01		
项目名称	开窗/分支支架腔内治疗非健康锚定区及短锚定区的主动脉夹层/动脉瘤的前瞻、单臂、单中心研究		
项目来源	自选课题		
研究单位	北京大学人民医院	承担科室	血管外科
主要研究者	李 伟	职 称	主任医师

审查类别	审查方式	审查日期
初始审查	快速审查	2017-12-11

审查地点	北京市西城区西直门南大街 11 号 北京大学人民医院医学伦理委员会
审查委员	杨拔贤
审查文件	1. 初始审查申请：2017-12-04<br>2. 研究方案：1.0，2017-10-10<br>3. 知情同意书：1.0，2017-10-10<br>4. 学术委员会科学研究委员会审评批件<br>5. 研究者简历

审查意见：作必要的修正后同意	
根据卫生部《涉及人的生物医学研究伦理审查办法（试行）》（2007）、CFDA《药物临床试验质量管理规范（2003）》、《药物临床试验伦理审查工作指导原则（2010）》、《医疗器械临床试验规定（2004）》、《体外诊断试剂临床试验技术指导原则（2014）》、WMA《赫尔辛基宣言》和CIOMS《人体生物医学研究国际道德指南》的伦理原则，经本伦理委员会审查，具体意见如下：<br>关于研究方案：<br>1. 依据“前瞻性观察性研究”的特点作通篇修改，并规范顺序编号；<br>2. 在正文中补充“研究随访计划、主要测量和评价指标”；<br>3. 简化“退出标准”的内容。<br>关于知情同意书：<br>1. 依据方案修正的内容作相应修正；<br>2. 删除文中与本研究无关的内容；<br>3. 重新书写“关于费用和补偿”项内容，建议修改为“本研究并不增加除常规医疗费用外的费用，也没有任何补偿”；<br>4. 建议将“报酬”项内容修改为“参加本研究没有任何报酬”。<br><br>按审查意见修改后的文件或对审查意见不同观点的陈诉，请提交“复审申请”。方案/知情同意书请注明新的版本号和版本日期并以阴影和/或下划线方式标注修改部分，报伦理委员会审查，经批准后执行。	
调整的年度/定期跟踪审查频率	NA
伦理委员会	北京大学人民医院医学伦理委员会 （盖章）
主任委员签字	[illegible]
日 期	2017-12-12.

首都医科大学附属北京友谊医院

实验动物管理与使用委员会

审查证明

项目名称：涡流通过上调血管内皮细胞 miR-126-3p/TGF-β 信号通路加速胸主动脉瘤扩张的机制研究

项目单位：首都医科大学附属北京友谊医院，血管外科

项目负责人：刘明远

本课题已经通过首都医科大学附属北京友谊医院实验动物管理与使用委员会的实验动物使用评审。

项目批准号：20-2007

实验动物中心主任：[illegible] 2022-08-29
